# An R-loop-initiated CSB–RAD52–POLD3 pathway suppresses ROS-induced telomeric DNA breaks

**DOI:** 10.1093/nar/gkz1114

**Published:** 2019-11-28

**Authors:** Jun Tan, Meihan Duan, Tribhuwan Yadav, Laiyee Phoon, Xiangyu Wang, Jia-Min Zhang, Lee Zou, Li Lan

**Affiliations:** 1 Massachusetts General Hospital Cancer Center, Harvard Medical School, Charlestown, MA 02129, USA; 2 Department of Radiation Oncology, Massachusetts General Hospital, Harvard Medical School, Boston, MA 02115, USA; 3 UPMC Hillman Cancer Center; 5117 Centre Avenue, Pittsburgh, PA 15213, USA; 4 Department of Pathology, Massachusetts General Hospital, Harvard Medical School, Boston, MA 02115, USA

## Abstract

Reactive oxygen species (ROS) inflict multiple types of lesions in DNA, threatening genomic integrity. How cells respond to ROS-induced DNA damage at telomeres is still largely unknown. Here, we show that ROS-induced DNA damage at telomeres triggers R-loop accumulation in a TERRA- and TRF2-dependent manner. Both ROS-induced single- and double-strand DNA breaks (SSBs and DSBs) contribute to R-loop induction, promoting the localization of CSB and RAD52 to damaged telomeres. RAD52 is recruited to telomeric R-loops through its interactions with both CSB and DNA:RNA hybrids. Both CSB and RAD52 are required for the efficient repair of ROS-induced telomeric DSBs. The function of RAD52 in telomere repair is dependent on its ability to bind and recruit POLD3, a protein critical for break-induced DNA replication (BIR). Thus, ROS-induced telomeric R-loops promote repair of telomeric DSBs through CSB–RAD52–POLD3-mediated BIR, a previously unknown pathway protecting telomeres from ROS. ROS-induced telomeric SSBs may not only give rise to DSBs indirectly, but also promote DSB repair by inducing R-loops, revealing an unexpected interplay between distinct ROS-induced DNA lesions.

## INTRODUCTION

Reactive oxygen species (ROS) induce multiple types of DNA damage, including oxidized bases, single-strand breaks (SSBs) and double-strand breaks (DSBs), throughout the genome (1). ROS arises from both endogenous and exogenous sources. Elevation of ROS levels is associated with cancer progression and treatment resistance (2). How cells respond to ROS-induced DNA damage is still incompletely understood. In particular, how cells repair ROS-induced DNA damage at telomeres is still largely unknown. Cancer cells use either telomerase or the Alternative Lengthening of Telomeres (ALT) pathway to extend telomeres (3). However, It is unknown how ROS-induced DNA damage is repaired in telomerase- and ALT-positive cancer cells.

DNA repair at telomeres is unique in many ways due to the repetitive nature of telomeric DNA, the presence of telomere-binding proteins, and the non-coding RNA TERRA. We have previously shown that XRCC1 is involved in the repair of ROS-induced SSBs at telomeres. The most deleterious form of ROS-induced DNA damage at telomeres is likely DSB, which could lead to a rapid loss of telomeres (4). How ROS-induced telomeric DSBs are repaired is not known. Non-telomeric DSBs are typically repaired by non-homologous end joining (NHEJ) and homologous recombination (HR) (5,6). The NHEJ pathway is inhibited at telomeres by multiple factors (7–9). Several HR proteins are involved in the maintenance of telomeres in ALT-positive cells. Furthermore, recent studies have implicated the break-induced DNA replication (BIR) pathway in the repair of replication stress or nuclease-induced DSBs at telomeres (10,11).

In this study, we investigated how ROS-induced DSBs are repaired at telomeres. We found that the efficient repair of ROS-induced telomeric DSBs requires the Cockayne Syndrome protein B (CSB) and RAD52. Both CSB and RAD52 are recruited to ROS-damaged telomeres by R-loops, which are induced by ROS in a TERRA- and TRF2-dependent manner in ALT-positive cells. Interestingly, ROS-induced SSBs are important for the accumulation of R-loops at damaged telomeres, suggesting an unexpected interplay between ROS-induced SSBs and the repair of ROS-induced DSBs. The binding of CSB to R-loops and its localization to damaged telomeres require its arginine 464. The recruitment of RAD52 to telomeric R-loops requires both CSB and the interaction of RAD52 with DNA:RNA hybrids through its lysine 144. At ROS-damaged telomeres, RAD52 uses its tyrosine 65 to interact with POLD3, a protein critical for BIR, and recruits POLD3. All of CSB, RAD52 and POLD3, as well as the interactions among them, are important for the efficient repair of ROS-induced telomeric DSBs. Together, these results reveal a previously unknown CSB–RAD52–POLD3 axis that is triggered by ROS-induced telomeric R-loops to remove ROS-induced DSBs at telomeres.

## MATERIALS AND METHODS

### Cell culture, plasmids and siRNAs

U2OS, BJ, HeLa and 293 cells were cultured in Dulbecco's modified Eagle's medium (DMEM, Lonza) with 10% (vol/vol) fetal bovine serum (Atlanta Biologicals) at 37°C, 5% CO_2_. SAOS2 cells were cultured in McCoy's 5a medium supplemented with 10% FBS, 2 mM glutamine and 1% penicillin/streptomycin. MEF cells were cultured in DMEM with 15% (vol/vol) fetal bovine serum. pLVX-IRES-Puro KR-TRF1/RFP–TRF1, pEGFP-RAD52, HA-RNaseH wild type and HA-RNaseH D210N were used in this study. CSB fragments 1–336, 337–509, 510–960, 961–1399 and 1400–1493 were cloned into pEGFP-C1 and PLVX-IRES-Puro (Myc-tag) vectors using XhoI and NotI as digestion sites, respectively. The R464A, RRAA, 3RA, K470A and K472A mutants in the CSB 337–509 (CSB-AD) fragment were created using overlapping PCR strategy. The PCR primers for cloning are summarized in Supplementary Table S1. CSB fragments stably expressing cell line was obtained by infection with pLVX-IRES-Puro CSB fragment lentivirus in CSB KO cell, and cells were selected with 1 g/ml Puromycin (Hyclone). Plasmids were transfected with Lipofectamine2000 (Thermo Fisher Scientific) using a standard protocol. siRNAs were transfected with Lipofectamine RNAiMax (Thermo Fisher Scientific) 48–72 h before analysis. The siRNAs used in this study were siCSB (SR320072, Origeneor), siRAD52 (gs5893,Qiagen), siPOLD3 (11) and siTRF2(sc-38505 Santa Cruz).

### KR activation

KR activation was described in our previous study (12). Briefly, U2OS cells were cultured in 35 mm glass-bottom dishes (MatTek, P35GC-1.5-14-C) at 70% confluence 24–36 h before the transfection. Light-induced KR activation was done by exposing cells to a 15 W Sylvania cool white fluorescent bulb for 30 min. Cells were recovered at indicated time before fixation. For FOK1-TRF1 induced damage, cells were transfected with FOK1-TRF1 plasmid and incubated for 24 h before harvest.

### Immunofluorescence staining and microscopy

For immunofluorescence staining, all cells were fixed with 3.7% (vol/vol) formaldehyde for 15 min at room temperature and washed three times with PBS. The cells were then permeabilized with 0.2% Triton X-100 for 10 min at room temperature and then washed with PBS twice. Next, the cells were blocked in 5% BSA (diluted in 0.05% PBST) for 1 h at room temperature. Primary antibodies were diluted in 5% BSA (0.05% PBST) and incubated overnight at 4°C. The cells were washed three times with 0.05% PBST and incubated with secondary antibodies diluted in 5% BSA for 1 h at room temperature, including Alexa Fluor 405/488/594 goat anti-mouse/rabbit/chicken IgG conjugate (1:1000). For S9.6 staining, the cells were fixed and permeabilized in a 35 mm glass bottom dish using a standard protocol, then incubated in buffer (10 mM Tris–HCl, 2 mM EDTA, pH 9) and steamed on a 95°C heating block for 20 min to expose the antigen. Then the dish was cooled, washed three times with PBS, and blocked in 5% BSA (diluted in 0.05% PBST). For the staining of POLD3 in RAD52 KO cells with expressing exogenous RAD52 WT or mutants, the GFP and KR were quenched by 2.5 M HCl and confirm it under microscope after fixing cells with 4% PFA, and then washed with TE buffer (pH 9) before permeabilizing cell with 0.2% Triton X-100. Primary antibodies used in this research were: anti-KR (1:1000, Ab961, Evrogen), anti-XRCC1(1:500, ab1838), anti-pAR (1:500, MABc547), anti-RFP (1:1000, Abcam ab62341), anti-DNA–RNA Hybrid [S9.6] (1:200, Kerafast, ENH001), anti-FLAG (1:400, F7425, Sigma), anti-H2AX (1:400, Millipore 05636), anti-Myc (1:200, Abcam, ab32), anti-GFP(1:400, Abcam, ab13970), anti-TRF1 (1:200, Santa Cruz, sc-56807), anti HA (1:200, Abcam ab9110) and anti-POLD3 (1:200, Abcam, ab182564).

The images were acquired using the Olympus FV1000 confocal microscopy system (Cat. F10PRDMYR-1, Olympus) and FV1000 software. For quantification of the percentage of foci-positive cells at sites of KR-TRF1, 15 cells were counted in every experiment and representative data are shown. For quantification of relative foci intensity, the intensity of foci and background was acquired by ImageJ 1.50i software and the fold increase of foci is calculated as the foci intensity divided by background intensity. The error bars represent SD and the *P*-value was calculated by Student's *t*-test. *P < 0.05, **P < 0.01, ***P < 0.001.

### TERRA knockdown and FISH

U2OS cells were grown to 70% confluency and then transfected with LNA gapmer oligos at a concentration of 4 mM in 100 ul Opti-MEM with 2 ul lipofectamine RNAiMAX (Thermo Fisher Scientific). A total of 2 ml of DMEM with 10% FBS was added to the cells after 2 h. For TERRA FISH, cells were fixed with 4% paraformaldehyde and then treated with 70% ethanol at 4°C for 2 h. DNA oligo probes (Supplementary Table S2) for RNA–FISH were mixed to reach a final concentration of 0.5 pmol/ml in hybridization solution (50% formamide, 2× SSC, 2 mg/ml BSA, 10% dextran sulfate-500K). Hybridization was carried out at 37°C overnight for RNA-FISH. The slides were washed three times with 2× SSC/50% formamide for 5 min at 44°C, followed by two more washes with 2× SSC for 5 min at 44°C. TERRA and Sense LNA gapmers were designed and synthesized by Exiqon (www.exiqon.com) with modified LNA bases and phosphothiolated backbone modification.

### Northern dot blot

All oligo probes were labeled with a 5′-oligonucleotide end labeling kit (Vectorlabs) and a maleimide-IR800 probe (LI_COR Bioscience). Total RNA was extracted using RNA mini Kit (Invitrogen, 12183018A). Total RNA (2 mg) was dot blotted on Nylon membrane. And membrane was UV crosslinked (1200 J). The membrane was hybridized with telomere probe (25 nM) in Ultrasensitive Hybridization buffer (Thermo Fisher Scientific, AM8669) at 42°C overnight. After hybridization, the membrane was washed with 2× SSC, 0.1% SDS (twice, 15 min each). And then wash the membrane with 2× SSC (twice, 15 min each). Use Bio-Rad to scan membrane for signal.

### CRISPR–Cas9 KO

The sgRNAs and methods for establishing CSB and RAD52 knockout cells were described in our previous work (13). Briefly, the sgRNAs were delivered to the cells through standard transfection. After 24 h, single cells were spread into 96-well plates or 10 cm dishes and grown for 10 days to obtain single colonies. The colonies were then transferred to 24-well plates and grown for ∼1 week prior to genome extraction, genotyping and western blotting verification. Western blotting gels of CRISPR KO cell verifications are displayed in Supplementary Figure S2.

### Colony formation assay

Cells that were transiently expressing KR-TRF1 were seeded into 60 mm Petri dishes (400 cells per dish). Cells were illuminated with or without 15 W white light for the indicated period until cells were attached. After 10 days, cells were fixed and stained with 0.3% crystal violet in methanol.

### β-Gal staining

KR-TRF1 was transiently transfected into U2OS and U2OS CSB KO cells and cultured without light for 36 h. All cells were treated with 15 W white light for 1 h and subsequently incubated for 7 days. Cellular senescence of ß-galactosidase was determined by applying cellular senescence staining kit's protocol (Cell Biolabs, CBA-230). Briefly, all cells in the 35 mm dish were washed 3 times with PBS and fixed by adding 1× fixing solution for 5 min at room temperature. Next, the cells were washed three times with PBS. 2 ml of freshly prepared Cell Staining Working Solution was used to completely cover the cells and incubated overnight at 37°C. Cell Staining Working Solution was removed and the stained cells were washed twice with 3 ml of 1× PBS. The cells were then stored in 1× PBS until further use.

### Metaphase chromosome spreads and telomere-PNA FISH analysis

KR-TRF1 was transiently transfected into cells and cultured without light for 36 h. All cells were treated with 15 W white light for 1 h and subsequently incubated for. days. The cells were then arrested at metaphase after treating them with 0.1 μg/ml colcemid (Sigma) for 3 h at 37°C. All cells were harvested through gentle pipetting, washed once with 1× PBS and incubated in 0.075 M KCl at 37°C for 30 min. After incubation, cells were fixed by adding fresh fixative (3:1 methanol/glacial acidic acid) and 0.075 M KCl (1:4 fixative/0.075 M KCl) for 10 min and centrifuged. The fixation process was repeated three times. Cells were then transferred onto wet slides and air-dried overnight in preparation for telomere-PNA FISH analysis.

All slides with chromosome spreads were fixed with 3.7% paraformaldehyde (Thermo Scientific) for 10 min after wash twice with PBS. Next, the slides were treated with RNase A (0.1 mg/ml) in PBS at 37°C and washed with PBS twice for 5 min each. The slides were then treated with 70%, 85%, 100% ethanol for 2 min each and dried for at least 30 min. After drying, 200 μl hybridization buffer (2% BSA 10 μl, 100 μg/ml yeast tRNA 1 μl, PNA-cy3 0.8 μl 125 nmol final, 0.6*SSC 20 μll, deionized formamide 140 μl, ddH2O 56.4 μL) was added onto the slide and covered with cover glass. All slides were heated at 85°C for 3 min to denature the DNA and then incubated at 37°C for 2 h. The slides were washed with wash buffer I (10 mM Tris, 70% formamide) twice for 15 min each and then washed with 0.1% PBST three times for 5 min each. The slides were air dried and sealed by using Fluroshield Mounting Medium with DAPI (Sigma, F6057).

### Telomere length assay

293T CSB KO cell lines were used in this study. Cells were cultured for over 30 passages. The total genomic DNA was purified using the GeneJET Genomic DNA Purification Kit (Thermo Scientific). Telomere restriction fragment analysis was performed using the TeloTAGGG Telomere Length Assay Kit (Roche) according to the manufacturer's protocol. Briefly, 2.5 g of genomic DNA was digested with Rsa I and Hif I restriction enzymes, separated on a 0.8% agarose gel and transferred to a nylon membrane. Transferred DNA was fixed on the membrane by UV-crosslinking (120 mJ) and hybridized with a DIG-labeled telomere probe, followed by incubation with anti-DIG-alkaline phosphatase and detection by chemiluminescence.

### U2OS CSB fragment stable cell construction

The method used to construct U2OS CSB fragment stable cells was described in our previous work (13). Briefly, the Myc-tagged CSB fragments in PLVX-IRES-Puro vectors were co-transfected with packaging plasmids into 293 FT cells for virus packaging. U2OS CSB KO cells were then cultured in the normal DMEM (10% FBS) and mixed with the virus at a 1:1 (vol/vol) ratio. After 48 h, the cells were further cultured in DMEM (10% FBS) with 1 μg/ml puromycin and the medium was changed once every 2 days.

### DNA:RNA hybrid-protein pull-down assay

The affinity of CSB -AD, ADC, ADN and ADC R464A to DNA: RNA hybrid (Supplementary Table S2) was determined using pierce magnetic RNA–protein pull-down kit. Briefly, pEGFP-C1-AD, ADC, ADN, AD-RRAA and ADC R464A were transfected to U2OS CSB KO cells. Cell lysates were prepared by adding lysate buffer (250 mM Tris–HCl PH7.4, 150 mM NaCl, 1 mM EDTA, 10% NP-40, 5% glycerol) into the cells. The target RNA included in the RNA–protein pull-down kit was labeled accordingly, added to magnetic beads in a 1.5 ml microcentrifuge tube and were washed with PH7.5 Tris–HCl. A master mix of RNA–protein binding was prepared to a final volume of 100 μl volume (10× protein–RNA binding buffer 10 μl, 50% glycerol 30 μl, protein lysate 1–30 μl, nuclease-free water to 100 μl). 100 μl of Master Mix was added to the RNA-bound beads and incubated for 30–60 min at 4°C with agitation. The supernatant was removed and stored for western blot analysis.

### Co-immunoprecipitation and western blots

Flp-in 293 and Flp-in TREX KR-TRF1 293 cells were transfected with indicated plasmids by Lipofectamine 2000 (Invitrogen). For KR-TRF1 expression was induced in Flp-in TREX KR-TRF1 293 cells by tetracycline, 2 μg/ml, for 24 h. For damage induced interaction, cells were light exposed for 1 h and recovery for 3 h. For anti-Myc immunoprecipitation, 30 μl anti-c-Myc agarose beads were added to each lysate. For anti-green fluorescent protein (GFP) immunoprecipitation, 2 μg anti-GFP monoclonal antibody (11814460001, Roche), and 30 μl of G-Sepharose protein beads (GE Healthcare Bio-Sciences) were added to each lysate. Mixtures were incubated at 4°C overnight with rotation; the supernatant was removed and protein beads were washed four times using 0.4 ml of lysis buffer.

For western blotting analysis, samples were boiled at 95°C for 5–8 min in SDS loading buffer. Then they were subjected to electrophoresis in 10–12% SDS-polyacrylamide gels and transferred to the polyvinylidene difluoride membrane. The membranes were blocked with 5% non-fat milk in PBS for 1 h before being incubated with the primary antibody at 4°C overnight. The primary antibodies for western blotting used in this study are GFP (11814460001, Roche, 1:2000), anti-TRF1 (1:200,Santa Cruz, sc-56807), anti-TRF2 (1:1000, Abcam, ab13579), anti-CSB (1:1000, ab96089,Abcam), anti-RAD52 (1:1000, Abcam, ab18264), anti-Myc (1:1000, ab9106, Abcam), anti-POLD3 (1:1000, Abcam, ab182564) and β-Actin (8H10D10, Cell Signaling Technology, 1:10 000). Then the cells were washed three to four times with 0.1% PBST and incubated with horseradish peroxidase (HRP)-conjugated secondary antibody (1:10 000) for 1 h at room temperature. The membranes were washed in 0.1% PBST for four times before exposure. Chemiluminescent HRP substrate was purchased from Millipore (Catalog#: WBKLS0500). Images were acquired in a Bio-Rad Universal Hood II machine with ImageLab software.

### Protein purification

The method used to purified RAD52 was described in our previous work (13). Briefly, Rosetta cells harboring pET28b-RAD52 and RAD52 K144A that encode hRAD52 with a C-terminal6x-his tag were grown to an optical density of 0.6 and induced by adding 0.3 mM Isopropyl β-d-1-thiogalactopyranoside (IPTG) for 3 h at 30°C. Ten grams of overexpressed cell mass were lysed in lysis buffer [25 mM Tris–HCl (pH 7.5), 500 mM KCl, 1 mM EDTA, 10% glycerol, 1 mM DTT, 0.01% Igepal, 1 mM PMSF, and a mixture of protease inhibitors] and sonicated. The lysed sample was centrifuged for 1 h at 16 000 r.p.m. (∼60 000 × g). Cleared supernatant was diluted five times and loaded on Affiblue beads in T buffer [25 mM Tris–HCl (pH 7.5), 1 mM EDTA, 10% glycerol, 1 mM DTT, 0.01% Igepal] with 100 mM KCl. Using fast protein liquid chromatography, RAD52 protein was eluted by a gradient of 0–2.5 M of NaSCN in T buffer. Fractions containing RAD52 protein were pooled together and dialyzed against T buffer with 300 mM KCl, and then incubated with Ni-NTA agarose beads for 2 h. RAD52 protein was eluted by a gradient of 10–300 mM imidazole in T buffer. Fractions containing RAD52 protein were pooled together and dialyzed against T buffer with 300 mM KCl. Dialyzed RAD52 was concentrated and stored at –80°C for biochemical assays.

Glutathione *S*-transferase (GST)-tagged human CSB-ADC WT and ADC R464A was expressed in Rosetta 2 (Novagen) and purified with a glutathione sepharose 4B (GE Healthcare Life Sciences) column. *Escherichia coli* Rosetta 2 transformed with a plasmid expressing the GST-tagged human CSB-ADC and ADC R464A was grown at 37°C in 1 l lysis buffer medium with 100 mg ampicillin and 15 mg chloramphenicol overnight. IPTG (final concentration; 0.1 mM) was then added to induce expression and the culture incubated for 4 h at 37°C. Cells were collected, resuspended in extraction buffer (50 mM Tris–HCl pH 7.5, 0.1 M NaCl, 1 mM DTT and 1% Triton X-100) and sonicated. Removing the cell debris by centrifugation. The cell-free extract was loaded onto a glutathione sepharose 4B (GE Healthcare Life Sciences) column.

### Oligo substrate preparation and electrophoretic mobility shift assay

The 5′-end of the oligo 1 was labeled using a 5′-oligonucleotide end labeling kit (Vectorlabs) and a maleimide-IR800 probe (LI_COR Bioscience). RNA–DNA substrate was prepared by annealing oligos 1 and 2 and confirmed. Briefly, 5′end-labeled oligo 1 was mixed with oligo 2 in buffer H [90 mM Tris–HCl (pH 7.5), 10 mM MgCl_2_ and 50 mM NaCl], heat denatured, and annealed by slow cooling. Annealed substrates were separated by 10% native polyacrylamide gel electrophoresis (PAGE)–Tris–acetate–EDTA. The corresponding gel bands were excised and eluted. RNA–DNA hybrid substrate was confirmed by mobility in native PAGE, heat denaturation, and RNaseH treatment.

For electrophoretic mobility shift assay. 5′-End maleimide-IR800-labeled hybrid substrate was incubated with RAD52 in Buffer B [25 mM Tris–HCl (pH 7.5), 1 mM MgCl_2_, 1 mM DTT, 50 μg/ml BSA] with 50 mM NaCl for 15 min at 37°C. Reactions were loaded on 6% PAGE–TBE gel and resolved at 4°C. Gels were imaged using an Odyssey scanner (Li-Cor Biosciences) and quantified.

## RESULTS

### Oxidative damage triggers telomere R loop accumulation in a TERRA and TRF2-dependent manner

**Figure 1.**
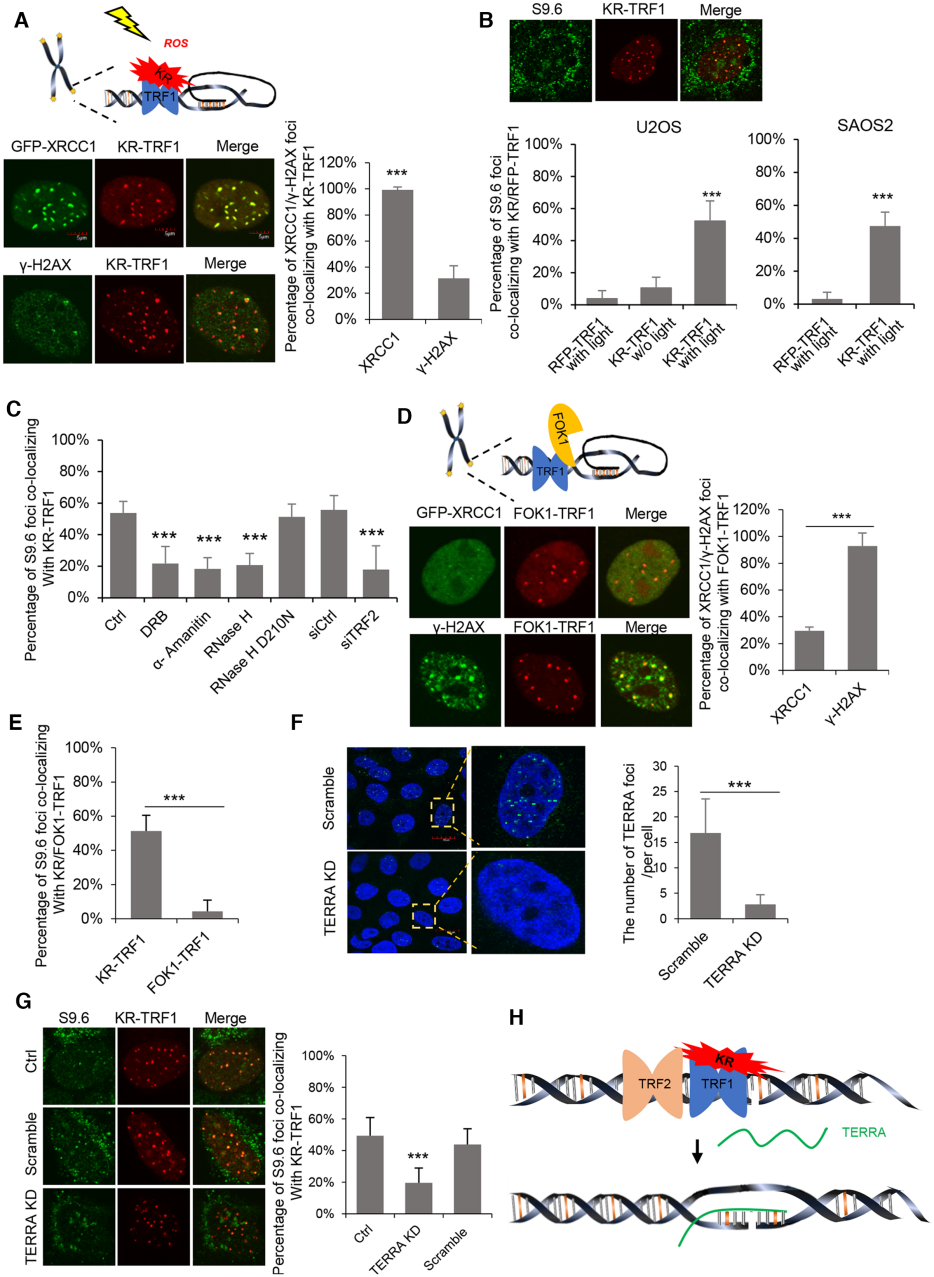
ROS induced SSB trigger R-loop formation at telomere dependently on TERRA and TRF2. (**A**) Schematic model of KR-TRF1. Co-localization of GFP-XRCC1 and γ-H2AX with KR-TRF1 in U2OS cells after exposing to light for 30 min are shown below the schematic model. The scale bars represent 5 μm. (**B**) The staining of S9.6 at KR-TRF1 and RFP-TRF1 after exposing to light for 30 min in U2OS cells and SAOS2 cells. (**C**) The staining of S9.6 at KR-TRF1 in U2OS cells after treating with DRB (20 μM, 24 h) and α-amanitin (100 μg/ml, 2 h), overexpressed with HA-RNaseH WT, D210N mutant, or TRF2 knockdown by siRNA. (**D**) Schematic model of FOK1-TRF1. The co-localization of GFP-XRCC1 and γ-H2AX with FOK1-TRF1 in U2OS cells after transfection are showed below the schematic model. (**E**) The staining of S9.6 at KR-TRF1 and FOK1-TRF1 in U2OS cells after exposing to light for 30 min and recovery for a half hour. (**F**) RNA FISH confirmed TERRA depletion in U2OS cells after 12 h of LNA treatment. Quantification of the number of TERRA signals in each cell. Error bar represents over 50 cells. (**G**) The staining of S9.6 at KR-TRF1 in U2OS cells after TERRA knockdown by LNA. (**H**) Schematic of R-loop formation after inducing ROS damage. For A–G, mean values with SD from 50 cells in three independent experiments are given. *P*-value is calculated by unpaired *t*-test. ***P < 0.001.

To investigate how telomeres respond to oxidative DNA damage, we have developed a strategy to introduce oxidative DNA damage locally at telomeres using a fusion protein of KillerRed (KR) and TRF1 (12). KR is a red fluorescent chromophore that releases ROS when activated by light. KR-TRF1 binds telomeres and, upon light activation, induces oxidative DNA damage, including oxidized bases, SSBs or DSBs at telomeres (12). Poly(ADP-ribose) polymerase (PARP) is a DNA nick sensor that binds to SSBs, where it synthesizes poly-ADP-ribose (PAR) at damage sites and facilitates the recruitment of XRCC1 (14). PAR and endogenous XRCC1 were efficiently recruited to almost all KR-TRF1-bound telomeres within 30 min after light activation (Supplementary Figure S1A). γ-H2AX, a marker of DSBs (15), was detected at a subset of KR-TRF1-bound telomeres in compared to XRCC1 (Figure 1A). This is consistent with the notion that ROS induce more SSBs than DSBs (16).

Previous studies suggested that SSBs induce R-loop formation efficiently *in vitro* (17). To test if KR-TRF1-induced damage leads to the accumulation of R-loops in ALT cells, we performed immuofluorence with the S9.6 antibody, which recognizes DNA: RNA hybrids (18). Compared with cells without induced oxidative damage at telomeres, U2OS cells expressing KR-TRF1 displayed a significant increase of telomere R-loops after light activation (Figure 1B and Supplementary Figure S1B). R-loop formation was confirmed in an additional ALT cell line SAOS2 (Figure 1B). However, we did not observe efficient R-loop formation at sites of KR-TRF1 in non-ALT cell lines, e.g. HeLa, BJ and MEF cells after damage (Supplementary Figure S1C). Although we cannot exclude that ROS induce low levels of R-loop in non-ALT cells, the induction of R-loops by ROS is much more robust in ALT cells. The R-loops signals at KR-TRF1-bound telomeres were reduced by expression of exogenous RNaseH1, an enzyme that cleaves the RNA in DNA:RNA hybrids, but not by the catalytically inactive RNaseH1-D210N mutant (Figure 1C and Supplementary Figure S1D). Furthermore, consistent with the idea that R-loops are formed during transcription, attenuation of transcription with RNA polymerase II inhibitor also reduced the formation of R-loops at damaged telomeres (Figure 1C and Supplementary Figure 1D).

Both SSBs and DSBs induce R-loops in transcribed regions (19,20). To investigate whether KR-TRF1-induced SSBs or DSBs are responsible for the R-loop induction, we tested the effects of the FokI–TRF1 fusion. The fusion of FokI endonuclease and TRF1 was used to induce DSBs at telomeres (10). Indeed, FokI–TRF1 induced more γ-H2AX but less XRCC1 foci at telomeres compared with KR-TRF1 (Figure 1D). However, the induction of telomere R-loops by FokI–TRF1 was much less efficient compared with KR-TRF1 (Figure 1E and Supplementary Figure S1E). These results suggest that SSBs are the primary R-loop-inducing oxidative damage at telomeres, although DSBs likely also contribute.

TERRA has been implicated in telomere R-loop formation and telomere maintenance in ALT-positive cells (21). To explore if TERRA is involved in the formation of damaged-induced R-loops, we partially depleted TERRA with a single-stranded antisense oligonucleotide (ASO) carrying locked nucleic acids (LNA) as previously described (Figure 1F and Supplementary Figure S1F) (22). Damage-induced telomere R-loop signals were reduced by the ASO-LNA targeting TERRA (Figure 1G), suggesting that telomere R-loops form in a TERRA-dependent manner in response to oxidative damage.

TRF2 plays an essential role in the maintenance of telomere integrity, and it promotes invasion of G-rich ssDNA overhang into the telomere duplex DNA to form t-loops (23). *In vitro* studies showed that TRF2 stimulated invasion of TERRA-like RNA into telomeric dsDNA, leading to R-loop formation (24). However, there is a lack of *in vivo* evidence for the function of TRF2 in R-loop formation. In cells expressing KR-TRF1, R-loop formation at telomeres decreased after TRF2 knockdown (Figure 1C and Supplementary Figure S1G). To test if TRF1 is also involved in R-loop formation, we used KR-TRF2 to induce telomere damage and knocked down TRF1 with siRNA. In contrast to TRF2 depletion, TRF1 knockdown did not reduce R-loops at damaged telomeres (Supplementary Figure S1H). Collectively, these results suggest that oxidative damage at telomeres induces R-loop formation in a TERRA- and TRF2-dependent manner (Figure 1H).

### CSB is recruited by telomeric R-loops to protect telomeres against oxidative damage

**Figure 2.**
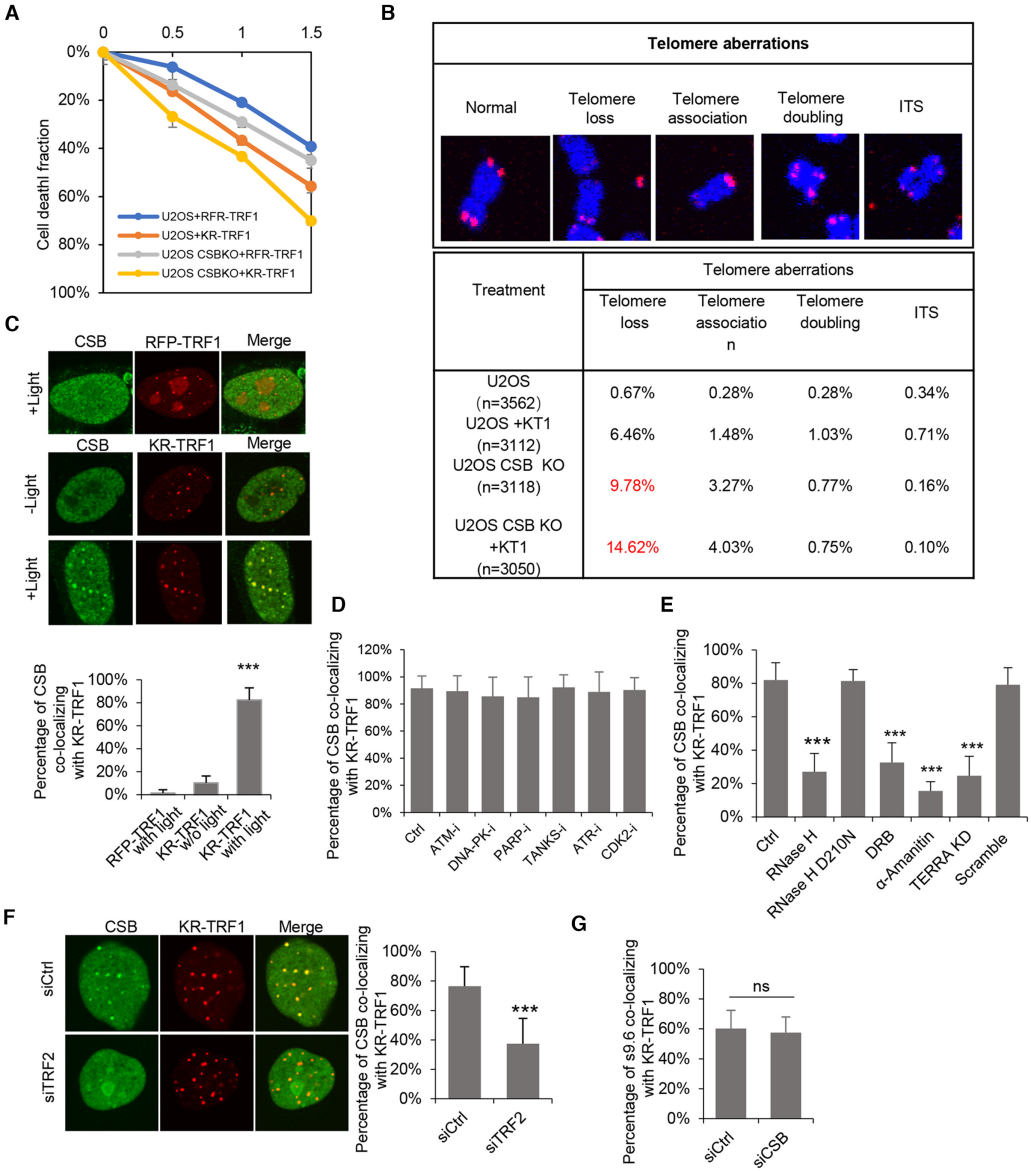
CSB is recruited to telomeric R-loops and contributes to cell survival and telomeric integrity. (**A**) Colony formation assays for U2OS and U2OS CSB KO cells with transiently expressing KR-TRF1 or RFP-TRF1. *n* = 3, error bars represent SD. (**B**) Telomere aberrations were found in U2OS and U2OS CSB KO cells with or without transiently expressing KR-TRF1. (**C**) Recruitment of CSB in KR-TRF1 or RFP-TRF1 expressing U2OS cells with or without light activation. Quantification of co-location frequency of myc-CSB and KR-TRF1/RFP-TRF1. (**D**) CSB foci co-localization with KR-TRF1 in U2OS cells treated with KU55933 (ATM inhibitor, 10 uM, 24 h), Nu7026 (DNA-PK inhibitor, 20 uM, 24 h), Olaparib (10 uM, 24 h), XAV-933(Tankyrase inhibitor,20 uM, 24 h), ATR inhibitor (10 uM, 24 h), and CDK2 inhibitor (5 uM, 24 h). (**E**) The staining of CSB at KR-TRF1 in U2OS cells after treating with overexpressed with HA-RNaseH WT, D210N mutant, DRB (20 μM, 24 h) and α-amanitin (100 μg/ml, 2 h), or TERRA knockdown by LNA. (**F**) CSB foci co-localization with KR-TRF1 in U2OS cells treated with TRF2 knockdown. (**G**) The staining of S9.6 at KR-TRF1 in U2OS cells after treating with CSB knockdown by siRNA. For (D)–(H), mean values with SD from 50 cells in three independent experiments are given. *P*-value is calculated by unpaired *t*-test, **P < 0.01, ***P < 0.001.

Our previous studies have suggested that CSB contributes to the repair of ROS-induced DSBs in actively transcribed regions (13,25). CSB was also implicated in telomere maintenance through a telomerase-mediated mechanism in telomerase-expressing cells (26). However, whether CSB is involved in telomere maintenance in ALT-positive cells is still unclear. To test if CSB is involved in the oxidative damage response at telomeres, we used CRISPR–Cas9 to knock out CSB in both telomerase-positive HEK293 cells and ALT-positive U2OS cells (Supplementary Figure S2A). CSB KO HEK293 cells exhibited short telomeres (Supplementary Figure S2B), which is consistent with a previous report (26). Importantly, CSB KO U2OS cells were more sensitive to telomeric oxidative damage compared with WT cells (Figure 2A). In the senescence assay using β-gal staining, CSB KO U2OS cells displayed an increase of senescence compared with WT cells, and this senescence phenotype was further increased by KR-TRF1-induced telomeric oxidative damage (Supplementary Figure S2C). The intensity of TRF1 foci at telomeres in CSB KO U2OS cells decreased after 3 months of passaging compared to wild-type U2OS (WT) cells (Supplementary Figure S2D). In metaphase spreading, telomere loss was increased from 9.78% to 14.62% in CSB KO cells by KR-TRF1-induced damage (Figure 2B). Of note, telomere shortening was observed in CSB deficient-Cockayne syndrome patients’ cells (26). Our results suggest that CSB is required for maintaining telomere length, cell survival, and telomeric integrity after ROS damage in ALT cells.

To elucidate the role of CSB in the telomeric oxidative damage response, we examined its localization in cells with or without telomere damage. CSB was detected at a small fraction of telomeres in cells without induced telomere damage, and the colocalization of CSB with telomeres was markedly increased after light activation of KR-TRF1 (Figure 2C). Inhibition of several proteins involved in telomere maintenance or the DNA damage response, including ATM, ATR, DNA-PKcs, PARP, Tankyrase and CDK2, did not affect the localization of CSB to damaged telomeres (Figure 2D). In contrast, RNA polymerase II inhibitors (DRB or α-amanitin) reduced the recruitment of CSB (Figure 2E and Supplementary Figure S2E). The localization of CSB to damaged telomeres was also reduced when R-loops were suppressed by TERAA ASO-LNA, RNaseH1 or TRF2 siRNA (Figure 2E and F, Supplementary Figure S2E and F). However, unlike TERRA and TRF2, CSB was not required for R-loop formation (Figure 2G and Supplementary Figure S2G). These results suggest that CSB is recruited to damaged telomeres by R-loops to protect telomeres against DNA damage.

### CSB R464 contributes to DNA:RNA hybrid binding

**Figure 3.**
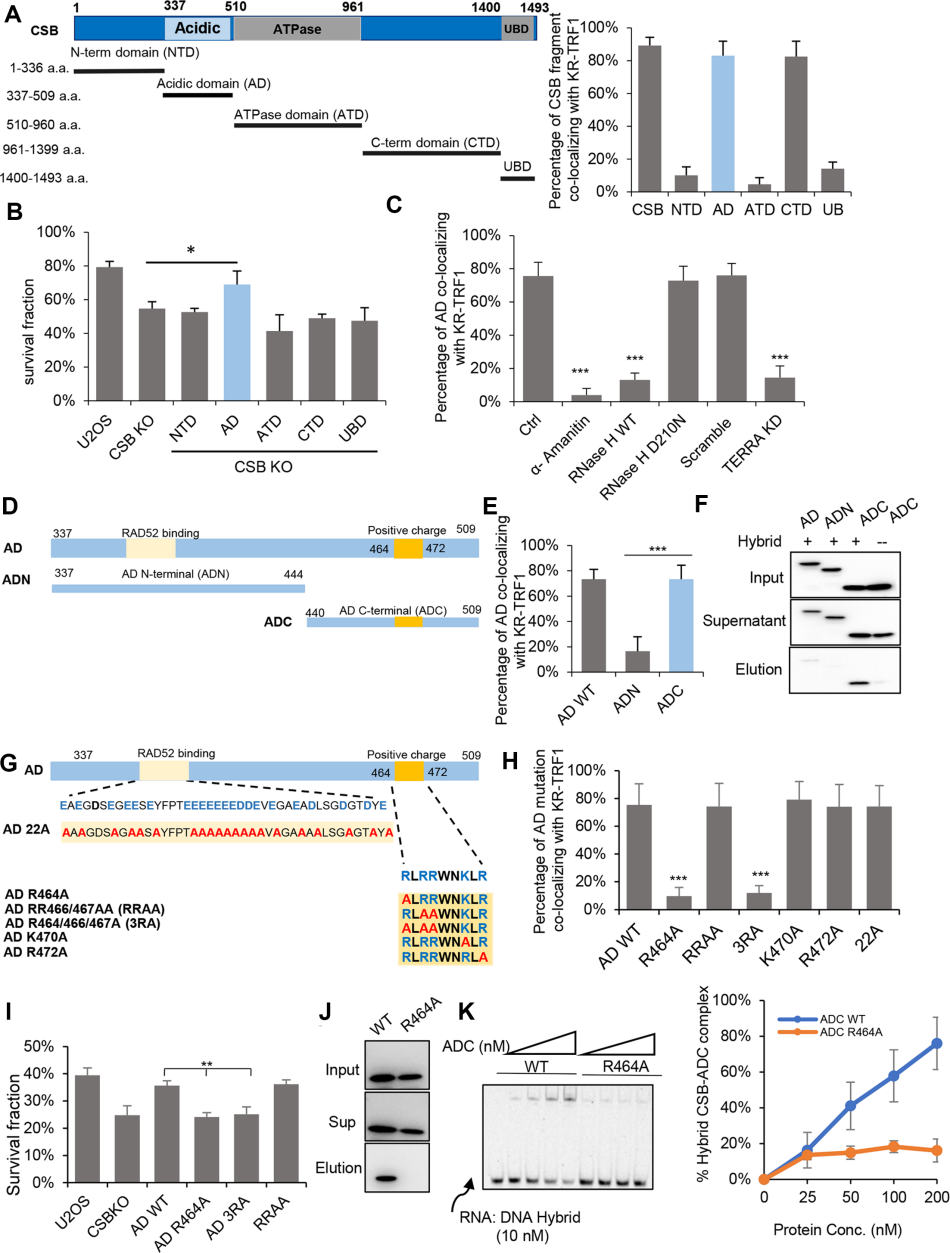
R464 of CSB contributes to DNA: RNA hybrid binding. (**A**) Schematic diagram of CSB fragments. The left panel is the quantification of co-location frequency of CSB fragments to KR-TRF1in U2OS cells. (**B**) Cell survival of CSB WT and KO cells expressing indicated fragments of CSB with 1 h light activation of KR-TRF1 in U2OS cells. *n* = 3, unpaired *t*-test, error bars represent SD, *P < 0.05 (**C**) GFP-AD foci co-localization with KR-TRF1 in U2OS cells treated with α-amanitin (100 μg/ml, 2 h), overexpressed with HA-RNaseH WT, D210N mutant or TERRA depleted with LNA. (**D**) Schematic diagram of CSB-ADN and ADC. (**E**) AD WT, ADN and ADC co-localization with KR-TRF1 after light activation. (**F**) The affinity of GFP-AD, GFP-ADN, and GFP-ADC to DNA: RNA hybrid by biotin-labeled hybrid pulldown using cell lysate from U2OS CSB KO cells. (**G**) Schematic diagram of CSB-AD 22A, R464A, RRAA, 3RA, K470A and R472A. (**H**) The recruitment of CSB-AD mutants to KR-TRF1 after light activation. (**I**) Colony formation assays for U2OS CSB KO cells expressing GFP-AD WT, AD R464A, AD3RA, and AD RRAA after inducing ROS damage by KR-TRF1 with 1.5 light activation. *n* = 3, unpaired t-test, error bars represent SD, **P < 0.01 (**J**) The affinity of GFP-ADC and GFP-ADC R464A to DNA: RNA hybrid by biotin-labeled hybrid pulldown using cell lysate from U2OS CSB KO cells. (**K**) DNA:RNA hybrids EMSA assay of purified CSB-ADC WT and R464A protein. For (C), (E) and (H), Mean values with SD from 50 cells in three independent experiments are given. *P*-value is calculated by unpaired *t*-test. ***P < 0.001.

To investigate the mechanism of how CSB is recruited to telomeric R-loops, we generated several fragments of CSB based on its structure (Figure 3A). The N-terminal domain (NTD), ATPase domain (ATD), and ubiquitin-binding domain (UBD) were not recruited to damaged telomeres. In contrast, the acidic domain (AD) and C-terminal domain (CTD) of CSB were recruited (Figure 3A and Supplementary Figure S3A). To understand how these fragments of CSB contribute to cell survival after oxidative damage, we stably expressed each CSB fragment in CSB KO U2OS cells and examined cell survival after telomeric oxidative damage using colony forming assay. Among these CSB fragments, only AD partially rescued cell survival in CSB KO cells (Figure 3B). The recruitment of AD to damaged telomeres was reduced by R-loop suppression via polymerase II inhibition (α-amanitin), TERRA depletion, or RNaseH1 expression (Figure 3C and Supplementary Figure S3B).

To understand how CSB AD functions, we further truncated AD into AD N-terminus (307–444 a.a., AD-N) and AD C-terminus (444–509 a.a., AD-C) (Figure 3D). Only AD-C was recruited to damaged telomeres (Figure 3E and Supplementary Figure S3D). Given that the recruitment of AD to damaged telomeres was dependent on R-loop formation, we examined the ability of AD, AD-N and AD-C to capture DNA:RNA hybrids from cell lysates. AD-C bound to DNA: RNA hybrid much more efficiently than AD-N (Figure 3F). The binding of full-length AD to DNA:RNA hybrids was lower than AD-C (Figure 3F). We noted that AD-N contains numerous acidic amino acids (13), which may reduce the affinity of AD-C to DNA: RNA hybrids.

From the amino acid sequence of AD-C, we identified a group of positively charged amino acids in 464–488 a.a. (464RLRRWNKLR488). We mutated some of these amino acids to alanines in full-length AD, resulting in AD mutants AD-R464A, -RR466/467AA (RRAA), -R464/466/467A (3RA), -K470A and -R472A (Figure 3G). AD-R464A, -3RA and AD-C-R464A mutants lost the ability to respond to telomeric oxidative damage (Figure 3H and Supplementary Figure S3C and S3E). In contrast, AD-RRAA, -K470A and -R472A mutants were recruited to damaged telomeres as efficiently as wild-type AD (AD-WT). Therefore, R464 is an amino acid critical for the affinity of AD-C to DNA: RNA hybrids. In addition, AD-R464A and -3RA did not rescue cell survival after damage as AD-WT and AD-RRAA did (Figure 3I). In pulldown assays, AD-C-R464A displayed a reduced affinity to DNA:RNA hybrids compared with AD-C (Figure 3J). In EMSA, purified CSB-ADC-R464A displayed reduced affinities to DNA:RNA hybrid (Figure 3K and Supplementary Figure S3F).Collectively, these results suggest that the R464 of CSB is critical for R-loop binding, which is required for the localization and function of CSB at damaged telomeres.

### The CSB-RAD52 axis promotes repair of ROS-induced telomeric DSBs

**Figure 4.**
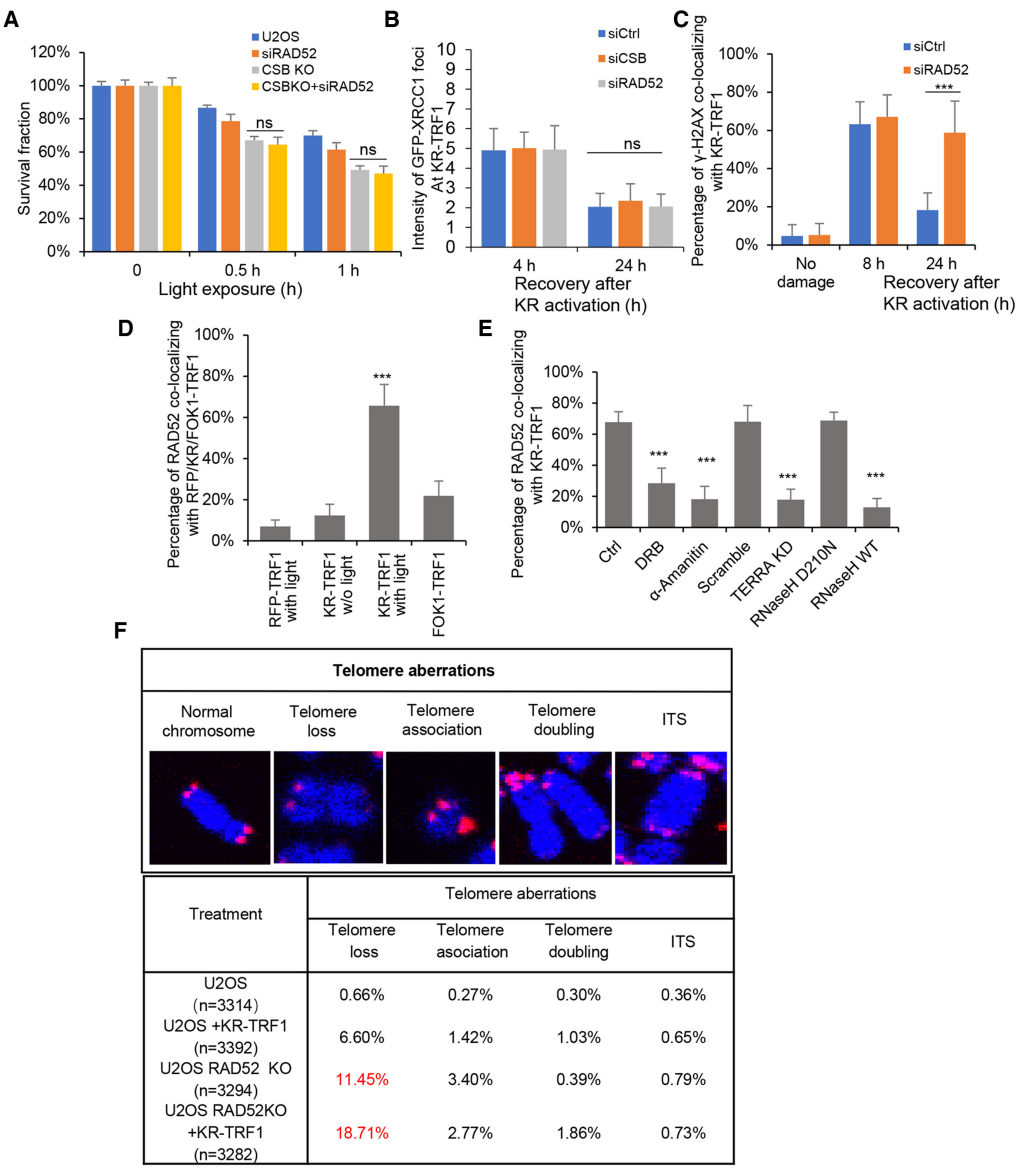
CSB-RAD52 cascade contributes to the repair of ROS induced telomeric DSB. (**A**) Colony formation assays for U2OS CSB KO cells with RAD52 knockdown by siRNA transiently expressing KR-TRF1. (**B**) The intensity of GFP-XRCC1 foci after CSB or RAD52 knockdown by siRNA at 4h and 24h after light activation. (**C**) The clearance of γ-H2AX after RAD52 knockdown. (**D**) The recruitment of RAD52 to RFP/KR/FOK1-TRF1after light activation or transfection. (**E**) The recruitment of RAD52 to KR-TRF1 after treating with DRB (20 μM, 24 h) and α-amanitin (100 μg/ml, 2 h), TERRA knockdown by LNA, or overexpressed with HA-RNaseH WT, D210N mutant. (**F**) Telomere aberrations were found in U2OS and U2OS RAD52 KO cells with or without transiently expressing KR-TRF1. For (B), (C), (D) and (E), Mean values with SD from 50 cells in three independent experiments are given. *P*-value is calculated by unpaired *t*-test. ***P < 0.001.

RAD52 functions downstream of CSB in the repair of ROS-induced DSBs in transcribed regions (13). RAD52 is also involved in telomere maintenance in response to replication stress and in ALT-positive cells (11,27). In cells expressing KR-TRF1, both CSB and RAD52 contributed to cell survival in response to telomeric oxidative damage (Figure 4A). Importantly, RAD52 knockdown did not further increase the sensitivity of CSB KO cells to telomere damage (Figure 4A), indicating that CSB and RAD52 function in the same pathway. The clearance of XRCC1 (a marker for SSB repair) at telomeres was not affected by CSB or RAD52 knockdown (Figure 4B). However, the clearance of γ-H2AX at telomeres was delayed by RAD52 depletion (Figure 4C and Supplementary Figure S4A). Consistent with the distinct roles of XRCC1 and CSB in the repair of telomeric SSBs and DSBs, knockdown of tankyrase, which is required for the recruitment of XRCC1 to damaged telomeres (28), did not affect the telomere localization of CSB (Figure 2D). Together, these results suggest that the CSB–RAD52 axis is important for the repair of ROS-induced DSBs.

The localization of RAD52 to telomeres was significantly stimulated by KR-TRF1 (Figure 4D and Supplementary Figure S4B). In contrast, FokI-TRF1 did not induce the telomere localization of RAD52 efficiently (Figure 4D and Supplementary Figure S4B). Given that both CSB and RAD52 bind DNA: RNA hybrids (13,29), we tested if the recruitment of RAD52 to damaged telomere is R-loop-dependent. Suppression of R-loops by several different means reduced the recruitment of RAD52 to damaged telomere (Figure 4E and Supplementary Figure S4C). When exposed to KR-TRF1-induced telomeric oxidative damage, RAD52 KO U2OS cells displayed: 1) increased sensitivity to telomeric damage (Figure 4A); 2) shorter telomeres (Supplementary Figure S4D); 3) increased senescence (Supplementary Figure S4E), and 4) increased telomere aberrations, including high levels of telomere loss (11.45%) and association (3.40%) (Figure 4F). Thus, while the CSB-RAD52 axis promotes the repair of ROS-induced telomeric DSBs, its activation may be primarily triggered by SSB-induced telomeric R-loops.

### RAD52 K144 contributes to DNA:RNA hybrid binding and localization to damaged telomeres

**Figure 5.**
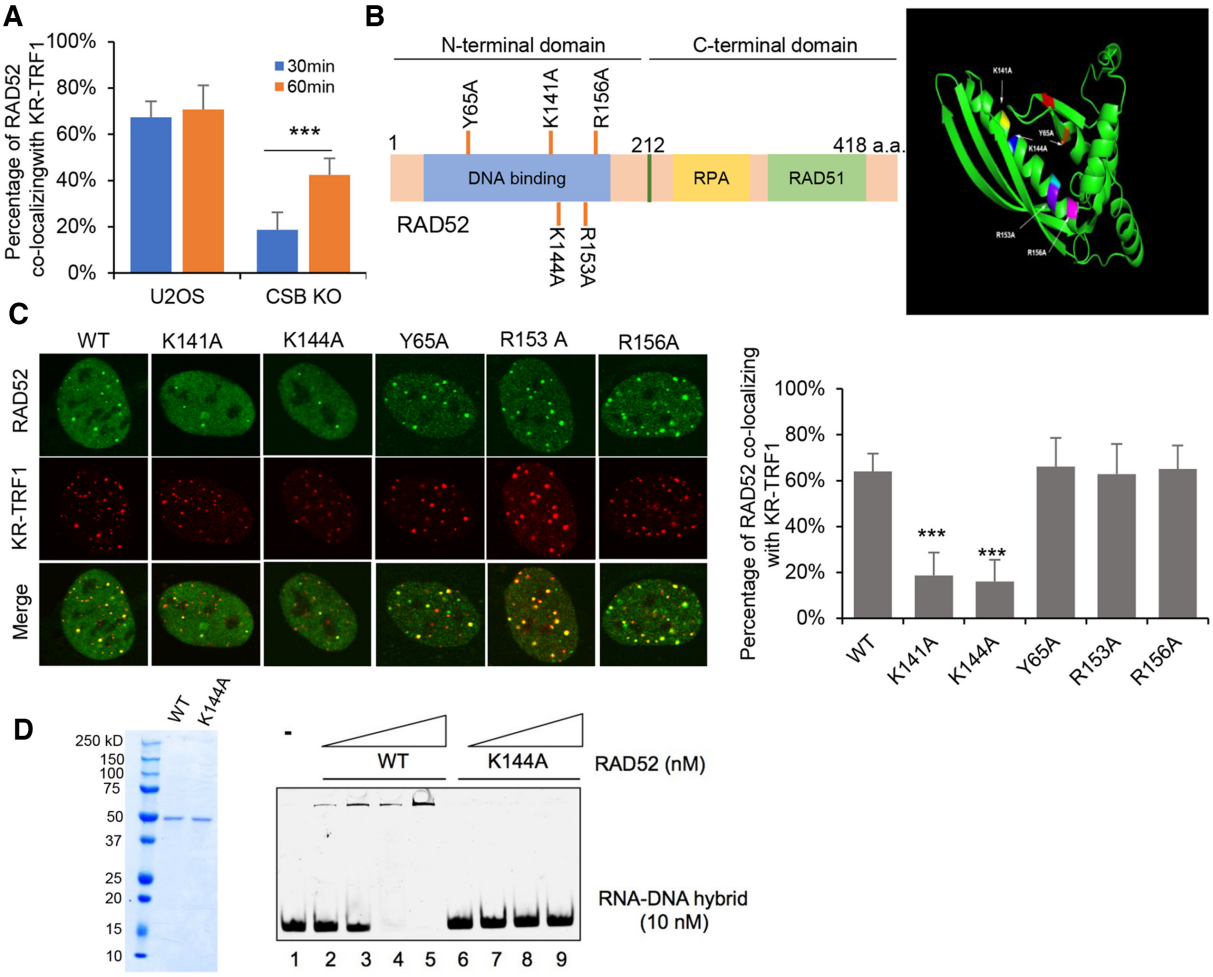
RAD52 is recruited to telomeric R-loop via CSB and K144-mediated hybrid binding. (**A**) The recruitment of RAD52 to damaged telomere in U2OS CSB KO cells after light exposure for 30 min and 60 min. (**B**) Schematic model of RAD52 structure and mutants. Right panel is Monomer structure of RAD52 and mutant sites. Y65A (orange), K141A (yellow), K144A (blue), R153A (purple), R156A (pink). (**C**) The recruitment of RAD52 mutants to KR-TRF1 after ROS damage. Quantification of the percentage of RAD52 co-localization with KR-TRF1. (**D**) DNA: RNA hybrids EMSA assay of purified RAD52 WT and RAD52 K144A protein. For (A) and (C), mean values with SD from 50 cells in three independent experiments are given. *P*-value is calculated by unpaired *t*-test. ***P < 0.001.

Although CSB promotes the recruitment of RAD52 to damaged telomeres, significant levels of RAD52 were recruited to damaged telomeres in CSB KO cells in a damage dose-dependent manner (Figure 5A and Supplementary Figure S5A). This result suggests that RAD52 localizes to damaged telomeres through both CSB-dependent and -independent mechanisms.

The N-terminal domain (NTD) of RAD52 is involved in the binding to ssDNA and dsDNA, whereas the C-terminal domain (CTD) is required for binding to RAD51 (30) and RPA (31). RAD52 also has affinities to RNA and DNA: RNA hybrids (29). We recently identified a KRRK motif (141–144) in RAD52 that is commonly found in other RNA-binding proteins (32). To test if this motif and other positively charged amino acids in the DNA-binding domain are involved in RNA and hybrid binding, we individually mutated Lys141, Lys144, Tyr65, Arg153 and Arg156 to alanines, resulting in RAD52 mutants RAD52-K141A, -K144A, -Y65A, -R153A and -R156A (Figure 5B). The positions of these amino acids were modeled in both RAD52 monomers and oligomers (Figure 5B and Supplementary Figure S5B). Among these RAD52 mutants, only RAD52-K141A and -K144A were not efficiently recruited to damaged telomeres (Figure 5C). Both RAD52-K141A and -K144A were recruited to laser-induced DSBs, indicating that these mutants retain some function in the general DSB response (Supplementary Figure S5C). In EMSA, purified RAD52-K144A displayed reduced affinities to ssDNA (Supplementary Figure S5D) and DNA:RNA hybrids (Figure 5D). Tyr65, Arg153, and Arg156 in the NTD have been reported to be critical for both ssDNA and dsDNA binding (33). However, mutations of these amino acids did not affect the R-loop-mediated RAD52 localization to damaged telomeres.

### RAD52 and POLD3-mediated BIR promotes repair of ROS-induced telomeric DSBs

**Figure 6.**
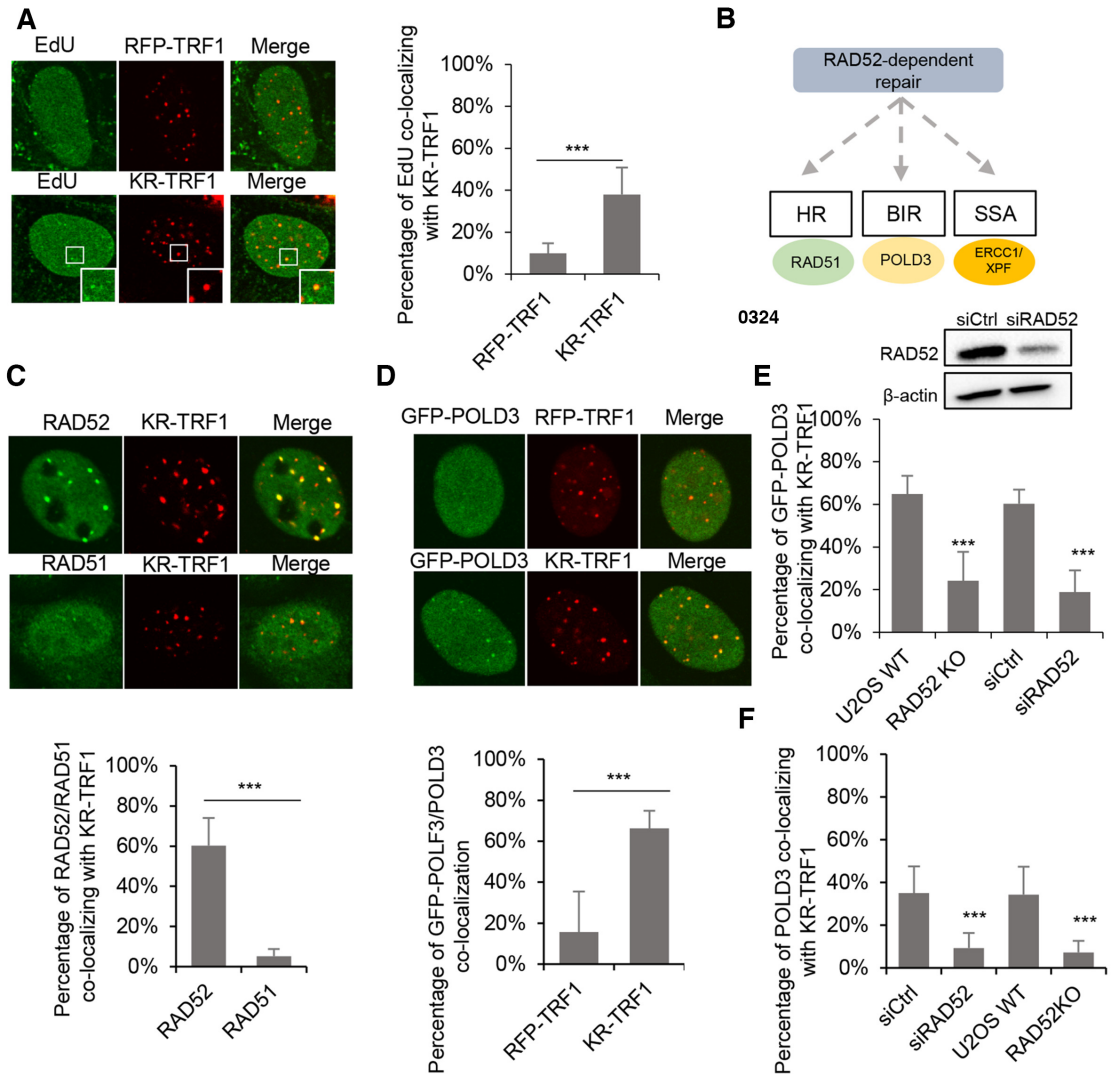
RAD52-POLD3 mediated BIR contributes to repair of ROS induced DSBs. (**A**) EdU incorporation after KR-TRF1-induced damage in U2OS. (**B**) Schematic of the RAD52 dependent repair pathway. (**C**) The recruitment of RAD52 and RAD51 to KR-TRF1 after light activation with 30 min recovery. (**D**) The recruitment of GFP-POLD3 to KR-TRF1 after light activation. (**E, F**) The recruitment of GFP-POLD3 (E) and POLD3 (F) to KR-TRF1 after RAD52 KO or knockdown by siRNA. For (A)–(F), mean values with SD from 50 cells in three independent experiments are given. *P*-value is calculated by unpaired *t*-test. ***P < 0.001.

To follow DNA repair synthesis at damaged telomeres, we monitored the EdU incorporation at telomeres bound by KR-TRF1 or RFP-TRF1, which is unable to release ROS. EdU incorporation at telomeres was significantly higher in KR-TRF1-expressing cells than in RFP-TRF1-expressing cells (Figure 6A). RAD52 is involved in HR, BIR, and single strands annealing (SSA) (Figure 6B). RAD51, which is critical for HR, was not efficiently recruited to KR-TRF1-damaged telomeres (Figure 6C). In contrast, FokI-TRF1-induced telomeric DSBs recruited RAD51 efficiently (Supplementary Figure S6A). Given that R-loop-CSB-RAD52 triggers rapid RAD51 recruitment at transcribed non-telomeric damage sites (13), our results indicate that R-loop–CSB–RAD52–RAD51 is not a primary repair pathway for telomeric DSBs. We do not exclude the involvement of R-loop–CSB–RAD52–RAD51 repair in the late stage of telomeric DSB repair. Indeed, we observed the recruitment of RAD51 24 hrs after damage induction (Supplementary Figure S6B). However, the late-stage recruitment of RAD51 is independent of CSB and RAD52 (Supplementary Figure S6B). In contrast to RAD51, POLD3, a subunit of DNA polymerase delta critical for BIR (34), was efficiently recruited to KR-TRF1-damaged telomeres (Figure 6D). Importantly, the recruitment of GFP-POLD3 and endogenous POLD3 was reduced by RAD52 loss (Figure 6E, F and Supplementary Figure S6C). SSA is initiated by DSB end resection and RAD52-mediated annealing of repetitive sequences flanking DSBs, followed by the removal of non-homologous 3′ ssDNA flaps by the ERCC1-XPF complex (35). In contrast to POLD3, neither ERCC1 nor XPF was localized to KR-TRF1-damaged telomeres (Supplementary Figure S6D). Therefore, R-loop–CSB–RAD52–POLD3 axis is likely to be the primary repair mechanism for telomeric DSBs after damage. HR-mediated DNA repair might play a role as a backup mechanism in the late stage of telomeric DSB repair.

### RAD52 Y65 is important for the recruitment of POLD3 and BIR

**Figure 7.**
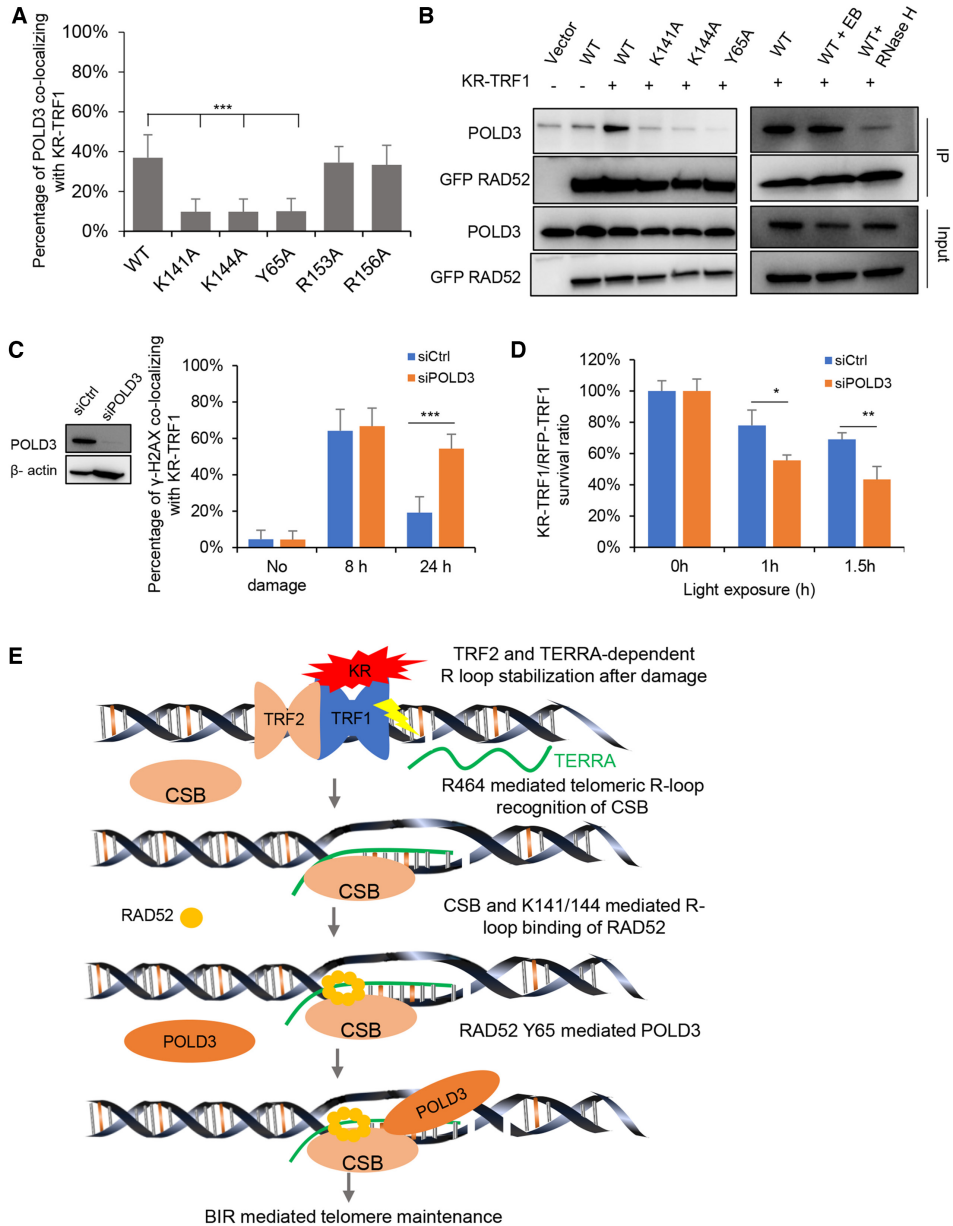
Tyrosine 65 (Y65) of RAD52 is required for the recruitment and the interaction with POLD3. (**A**) The recruitment of POLD3 to KR-TRF1 in U2OS RAD52 KO cells after transient expressing RAD52 mutants. (**B**) Co-immunoprecipitation of GFP-RAD52 WT and mutants with POLD3 after KR-TRF1-induced damage for 1 h (left). Co-immunoprecipitation of GFP-RAD52 WT with POLD3 after treating cell lysates with ethidium bromide (0.1 mg/ml) or RNase H (20 U/ml) for 30 min (right). (**C**) The clearance of γ-H2AX after POLD3 knockdown by siRNA. (**D**) Colony formation assays for U2OS and U2OS POLD3 knockdown by siRNA and transiently expressing KR-TRF1 or RFP-TRF1 with indicated time to light exposure. *n* = 3, error bars represent SD. (**E**) Schematic model of R-loop–CSB–RAD52 mediates BIR contribute to the repair of telomeric DSB induced by ROS. The current model is composed of the following sequential steps: ROS-induced R-loop formation is dependent on TERRA and TRF2; CSB–ADC–R464 recognizes R-loop; K141/144 mediates R-loop binding of RAD52; RAD52 R65 mediated POLD3 interaction promotes BIR. For (a) and (c), Mean values with SD from 50 cells in three independent experiments are given. *P*-value is calculated by unpaired *t*-test. ***P < 0.001.

Next, we monitored the recruitment of POLD3 to damaged telomeres in RAD52 KO cells expressing exogenous RAD52 WT or mutants. RAD52-Y65A, -K141A and -K144A did not rescue the recruitment of POLD3 to damaged telomere (Figure 7A and Supplementary Figure S7A). We have shown that RAD52-K141A and -K144A were unable to localize to damaged telomeres (Figure 5C). In contrast, RAD52-Y65A was able to localize to damaged telomeres but failed to recruit POLD3 (Figure 7A and Supplementary Figure S7A). Notably, in co-immunoprecipitation assays, RAD52-WT interacted with POLD3 in a damage-dependent manner in KR-TRF1-expressing cells (Figure 7B). Interestingly, the interaction between RAD52 and POLD3 were largely abolished by RNase H treatment, but not ethidium bromide (Figure 7B), suggesting that the interaction between RAD52 and POLD3 occur within the context of DNA: RNA hybrid at damaged telomeres. The interactions of RAD52-K141A, -K144A and -Y65A with POLD3 were reduced compared with RAD52-WT (Figure 7B). These results indicate that RAD52 K141/K144-mediated recruitment of RAD52 and RAD52 Y65-mediated recruitment of POLD3 to DNA:RNA hybrids at damaged telomeres contribute to complex stabilization. In U2OS cells treated with POLD3 siRNA, the clearance of γ-H2AX at KR-TRF1-damaged telomeres was delayed (Figure 7C and Supplementary Figure S7B), and cell survival was reduced (Figure 7D), showing that POLD3 is functionally important for repairing ROS-induced telomeric DNA damage.

## DISCUSSION

In this study, we show that the BIR pathway activated by ROS is triggered by telomeric R-loops dependently on TERRA. We found that KR-TRF1 is more efficient than FokI-TRF1 in inducing telomeric R-loops. This result raises the possibility that ROS-induced SSBs rather than DSBs are the main inducer of R-loops (Figure 7E). When telomeres are damaged by ROS, both SSBs and DSBs are induced. Telomeric SSBs, if not repaired, will give rise to DSBs indirectly during replication. Close SSBs on opposite DNA strands could also generate DSBs. Our results suggest that telomeric SSBs may not only potentiate DSB formation, but also promote DSB repair by inducing R-loops. This finding reveals a surprising crosstalk between distinct ROS-induced DNA lesions at telomeres.

BIR has been recently implicated in the repair of replication stress or nuclease-induced DSBs at telomeres (10,11). POLD3 KD leads to delayed repair kinetics and increased sensitivities to telomeric damage in U2OS cells (Figure 7C and D). However, ROS-induced BIR is different from the BIR pathway trigged by the FokI-TRF1 fusion (10). First, KR-TRF1 but not FokI-TRF1 induces R-loops robustly at telomeres. Second, FokI-TRF1 but not KR-TRF1 triggers efficient recruitment of RAD51 to telomeres. Finally, only the KR-TRF1-induced POLD3 localization to telomeres, but not that triggered by FokI-TRF1, is dependent on RAD52. Together, these findings suggest that ROS and nuclease-generated DSBs activate telomeric BIR through distinct mechanisms, although POLD3 is involved in both contexts (Figure 7E).

Once telomeric R-loops are induced by ROS, they are recognized by both CSB and RAD52 (Figure 7E). Our recent studies showed that CSB has a high affinity for DNA:RNA hybrids at transcribed damage site (13). In this study, we find that the R464 of CSB is critical for hybrid binding at telomeres. RAD52 is known to bind both CSB and DNA:RNA (13,29). We show that both CSB and the interaction of RAD52 with DNA: RNA hybrids are important for its localization to damaged telomeres and its function in telomere repair. The K141 of RAD52 is a key residue for hybrid binding. Together, these results have provided a clear picture of how ROS-induced telomeric R-loops are sensed by CSB and RAD52.

Importantly, the mechanisms by which CSB and RAD52 protect telomeric DSBs are different from transcribed damage sites. We have recently shown that KR-induced DSBs in transcribed non-telomeric regions are repaired through a CSB–RAD52–RAD51 pathway (13). Interestingly, ROS does not induce efficient recruitment of RAD51 to damaged telomeres at early stage. This is unlikely due to the inability of telomeres to recruit RAD51 as FokI-TRF1 induces robust localization of RAD51 to telomeres. It is possible that ROS-induced changes of certain telomere-binding proteins interfere with RAD51 recruitment. Alternatively, oxidized telomeric ssDNA may disfavor the recruitment of RAD51. In contrast, the CSB-RAD52 axis operating in non-telomeric regions, the CSB-RAD52 axis at telomeres recruits POLD3 through a specific interaction between the Y65 of RAD52 and POLD3 (Figure 7E). This recruitment of POLD3 likely promotes BIR at ROS-induced telomeric DSBs, enabling damaged telomeres to avoid abrupt shortening. The ROS-induced telomeric BIR probably might involve additional factors to be determined and examined.

Cancer cells bypass replicative senescence by using either telomerase or Alternative Lengthening of Telomeres (ALT) pathway, which extends telomeres through a recombination-mediated mechanism. In our study, we found ALT cell lines such as U2OS and SAOS2 displayed a significant increase of telomeric R-loops in response to ROS-induced DNA damage at telomeres. In contrast, ROS damage did not efficiently trigger R-loop formation in HeLa, BJ and MEF cells (Supplementary Figure S1C). ALT cancer cells display elevated levels of TERRA compared with telomerase positive cancer cells (36), leading to an increase of telomeric R-loops that facilitate recombination at telomeres. Given that R-loop formation is much more efficient in ALT cells than in non-ALT cells, our result suggest that the CSB––RAD52–POLD3 axis is preferentially utilized for telomere DNA repair in ALT cancers. The CSB-RAD52–POLD3-mediated telomeric BIR pathway discovered in this study is triggered by ROS through telomeric R-loops. Since ROS-induced R-loop formation is dependent on TERRA and TERRA is more abundant in ALT-positive cells (37), this ROS-induced BIR pathway may be activated more efficiently in ALT-positive cells. However, it is still unclear whether this pathway is specific to ALT-specific cells. Given that TERRA is also detected in non-ALT cells, this pathway may operate at a low level independently of ALT. In future studies, it would be interesting to investigate whether this ROS-trigged CSB–RAD52–POLD3 pathway is similar to or distinct from the natural ALT pathway. Furthermore, it would be important to explore whether blockage of this pathway can selectively kill cancer cells suffering from high levels of ROS.

## DATA AVAILABILITY

The datasets generated during and/or analyzed during the current study are available from the corresponding author upon request.

## Supplementary Material

gkz1114_Supplemental_File
