# Supplementary material for: An R-loop-initiated CSB–RAD52–POLD3 pathway suppresses ROS-induced telomeric DNA breaks
Source: Nucleic Acids Res. 2019 Nov 28;48(3):1285–300. doi: 10.1093/nar/gkz1114 (PMC7026659; doi:10.1093/nar/gkz1114)
Supplement: gkz1114_Supplemental_File [file gkz1114_supplemental_file.pdf]

An R-Loop-initiated CSB-RAD52-POLD3 Pathway Suppresses ROS-induced Telomeric DNA Breaks

Tan J. et.al

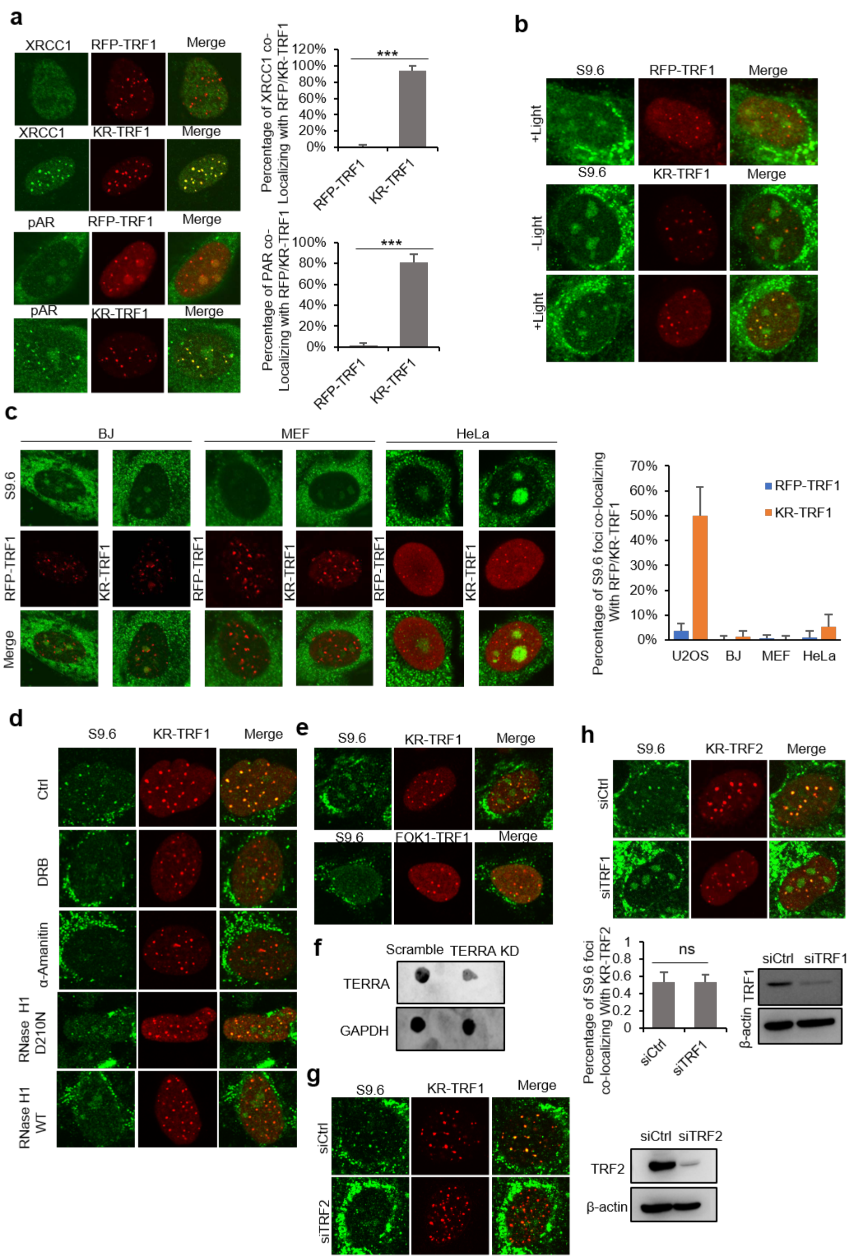

Supplementary Figure 1. TRF2 and TERRA, but not TRF1, is required for ROS induced R-loop formation at the telomere.

(a-b) The staining of XRCC1 (a), pAR (a) and S9.6 (b) with Abs at KR/RFP-TRF1 in U2OS cells after light exposure for 30 min and recovery for 30 min. **(c)** The staining of S9.6 at KR-TRF1 and RFP-TRF1 after exposing to light for 30 min and recovery for 30 min in BJ, MEF and HeLa cells. **(d)** The staining of S9.6 at KR-TRF1 after treating with DRB (20 μM, 24 h) and α-amanitin (100 μg/ml, 2 h) in U2OS cells overexpressing HA-RNaseH WT or D210N mutant. **(e)** The staining of S9.6 at KR-TRF1 and FOK1-TRF1 in U2OS cells after light treatment. **(f)** Northern dot blot after TERRA KD with LNA in U2OS cells. **(g)** The staining of S9.6 at KR-TRF1 in U2OS cells after treating with TRF2 knockdown by siRNA. **(h)** The staining of S9.6 at KR-TRF2 in U2OS after TRF1 knockdown by siRNA. For (a, c, h), Mean values with SD from 30 cells in three independent experiments are given. P-value is calculated by unpaired t-test.

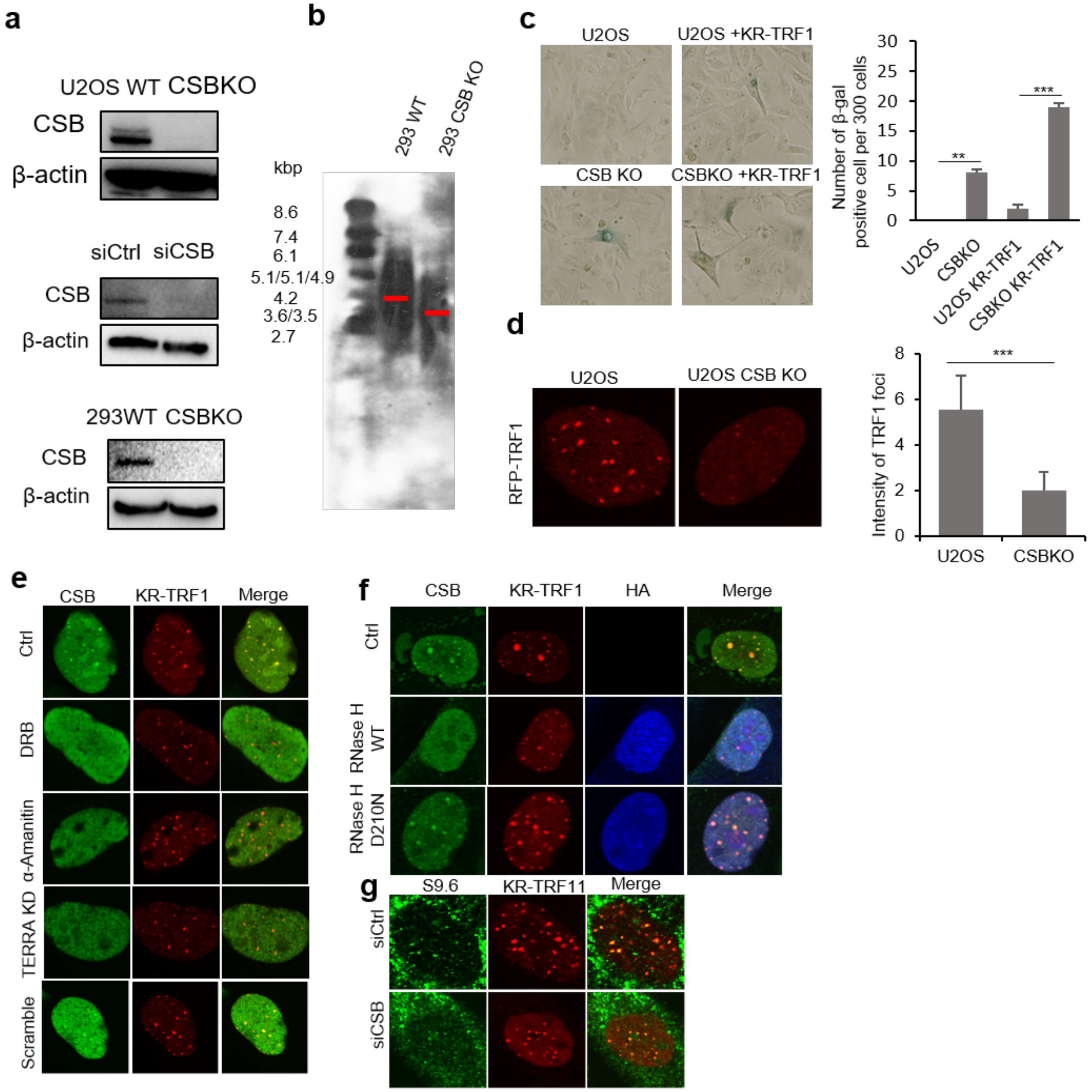

Supplementary Figure 2. CSB knockout leads to telomere shortening. (a) Western blot confirmed the CSB KO and KD efficiency in U2OS and 293 cells. **(b)** TRF analysis of average telomere length in 293 CSB KO cells after culturing for more than three months. **(c)** SA-β-gal staining of KR-TRF1 transiently-expressing in U2OS and U2OS CSB KO cells. Number SA-β-gal positive cells of per 300 cells were obtained from three independent experiments. Error bars represent SD, **P < 0.01, ***P < 0.001. **(d)** KR-TRF1 foci in U2OS CSB KO cells after culturing for more than three months. Quantification of the intensity of KR-TRF1 foci. The mean values of over 100 RFP-TRF1 foci with SD are showed. **(e-f)** The staining of CSB at KR-TRF1 in U2OS cells after treating with DRB (20 µM, 24 h) and α-amanitin (100 µg/ml, 2 h) and TERRA knockdown by LNA (D), overexpressed with HA-RNaseH WT, D210N mutant). **(g)** The staining of S9.6 at KR-TRF1 in U2OS cells after treating with CSB knockdown by siRNA.

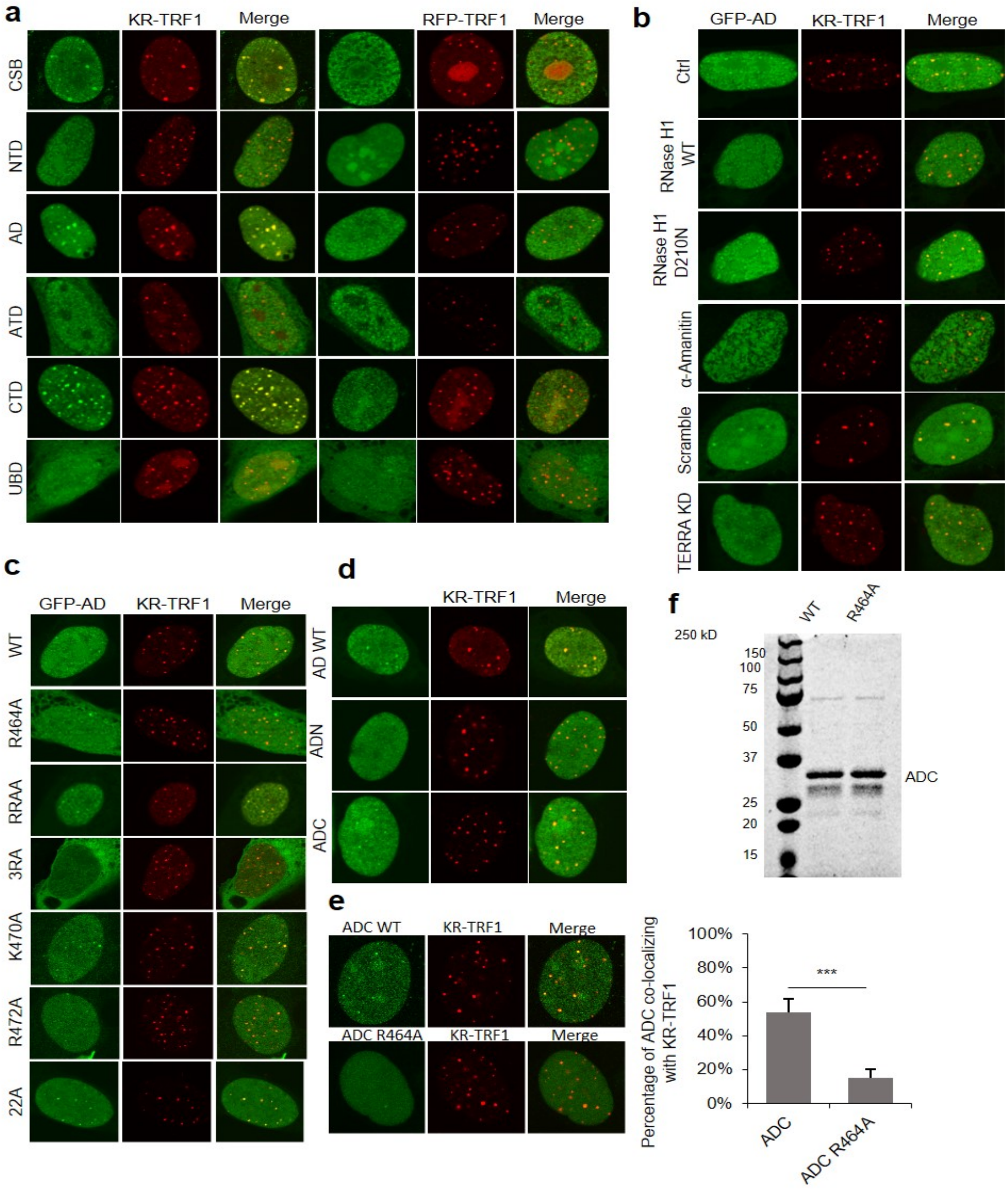

Supplementary Figure 3. R464 of CSB contributes to DNA: RNA hybrid binding. (a) The recruitment of CSB fragment to KR-TRF1/RFP-TRF1 after light activation**. (b)** GFP-AD foci co-localization with KR-TRF1 in U2OS cells treated with α-amanitin (100 µg/ml, two h), overexpressed with HA-RNaseH WT, D210N mutant or TERRA depleted with LNA**. (c)** The recruitment of GFP-AD mutant to KR-TRF1. **(d)** GFP-AD WT, ADN and ADC co-localization with KR-TRF1 after light activation. **(e)** The recruitment of ADC WT and ADC R464A to KR-TRF1. **(f)** Coomassie blue staining of GST-ADC WT and R464A.

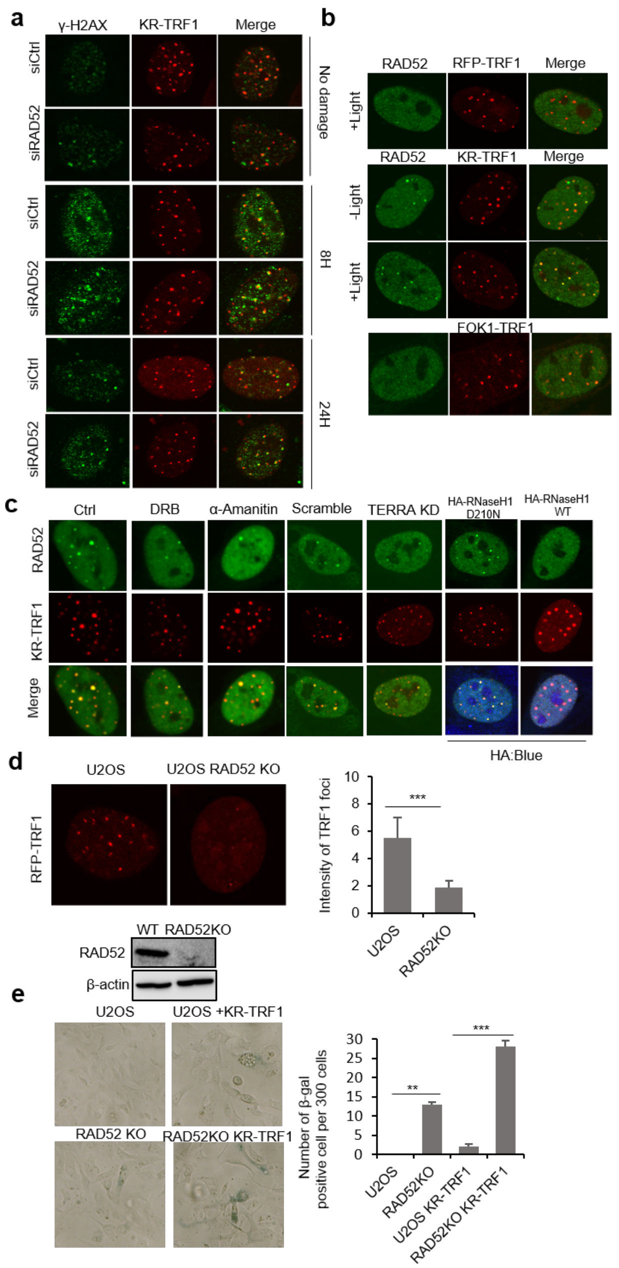

Supplementary Figure 4. RAD52 is recruited to telomeric R-loop. (a) The clearance of γ-H2AX after RAD52 KD by siRNA. **(b)** The recruitment of RAD52 to RFP/KR/FOK1-TRF1after light activation or transfection. **(c)** The recruitment of RAD52 to KR-TRF1 after treating with DRB (20 μM, 24 h) and α-amanitin (100 μg/ml, 2 h), TERRA knockdown by LNA, or overexpressed with HA-RNaseH WT, D210N mutant. **(d)** TRF1 foci in U2OS RAD52 KO cells after culturing for more than three months. Quantification of the intensity of KR-TRF1 foci. Error bar represents over 100 KR-TRF1 foci. **(e)** SA-β-gal staining of KR-TRF1 transiently-expressing in U2OS and U2OS RAD52 KO cells. Number SA-β-gal positive cells (n = 300) were obtained from three independent experiments, Error bars represent SD, $**P < 0.01$, $***P < 0.001$.

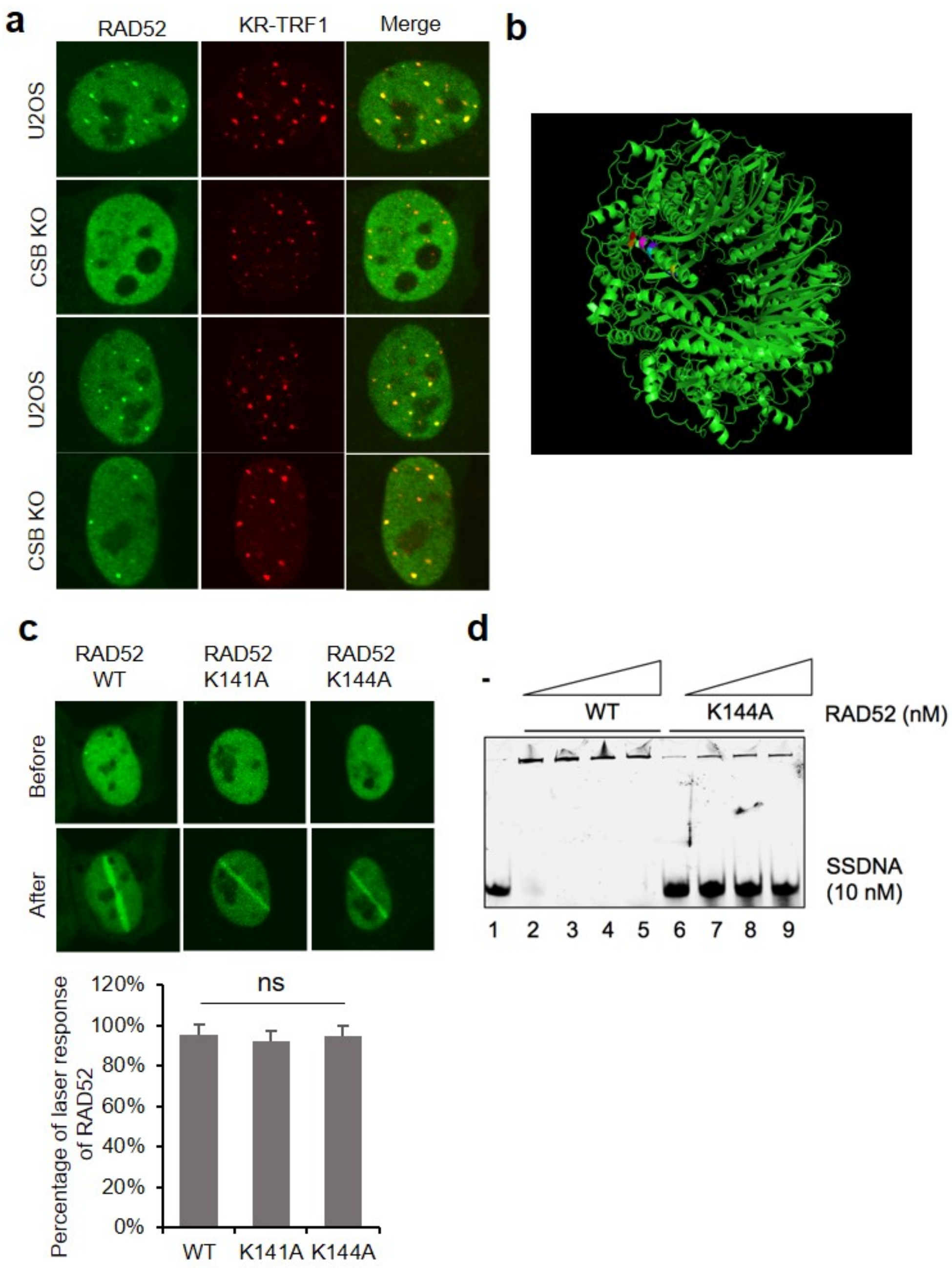

Supplementary Figure 5. The recruitment of RAD52 to damaged telomere is dependent on CSB and DNA: RNA binding. **(a)** The recruitment of RAD52 to damaged telomere in U2OS CSB KO cells after light exposure for 30 min and 60 min. **(b)** Polymer structure of RAD52. **(c)** U2OS cells expressing with GFP-RAD52 K141A or K144A were laser-micro-irradiated and monitored by live cell imaging. **(d)** ssRNA EMSA assay of purified RAD52 WT and RAD52 K144A protein.

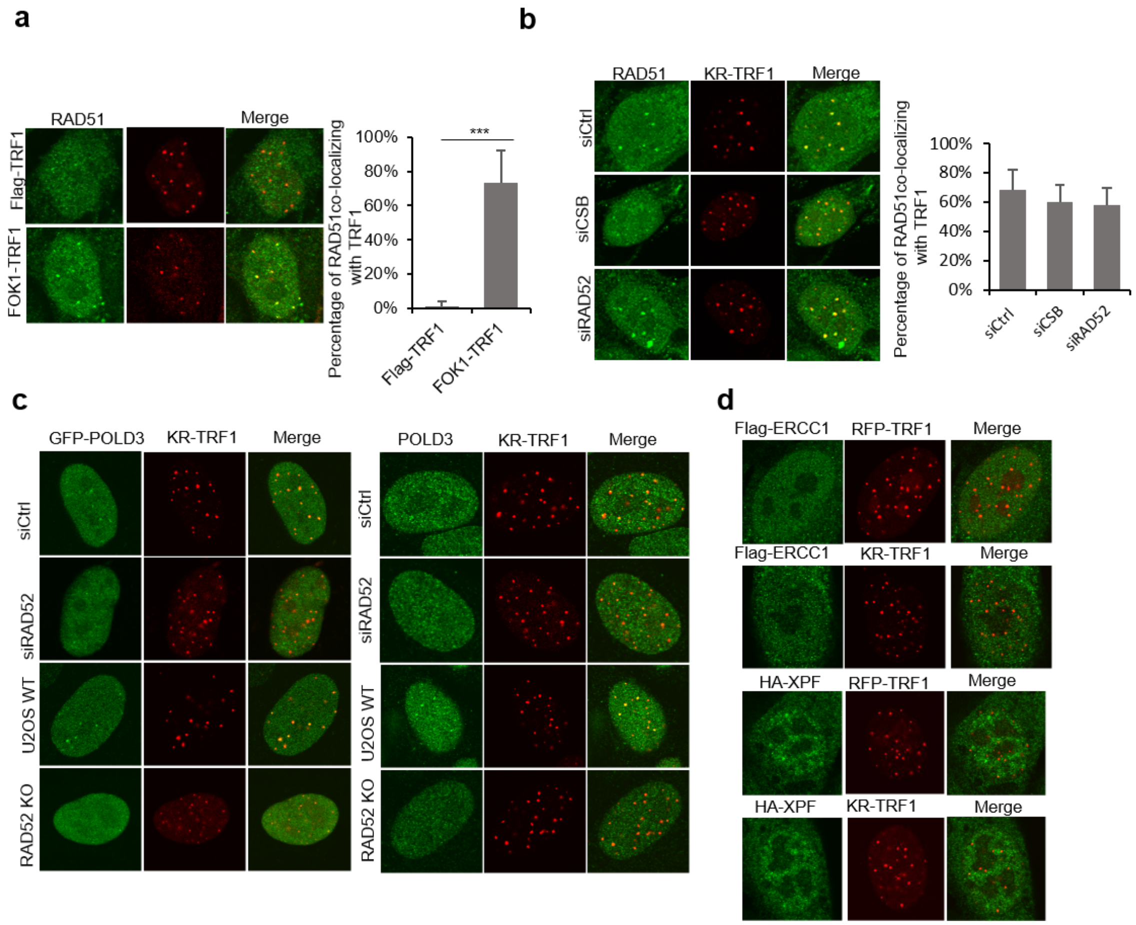

Supplementary Figure 6. The recruitment of POLD3 to damaged telomere requires RAD52.

(a) The recruitment of RAD51 to FOK1-TRF1 after transfection. **(b)** The recruitment of RAD51 to KR-TRF1 after inducing damage for 30 min and recovery for 24 hours in U2OS cells with CSB or RAD52 knockdown by siRNA. **(c)** The recruitment of POLD3 to KR-TRF1 in U2OS RAD52 KO cells after transient expressing RAD52 mutants. **(d)** The recruitment of ERCC1 and XPF to KR-TRF1 after light activation.

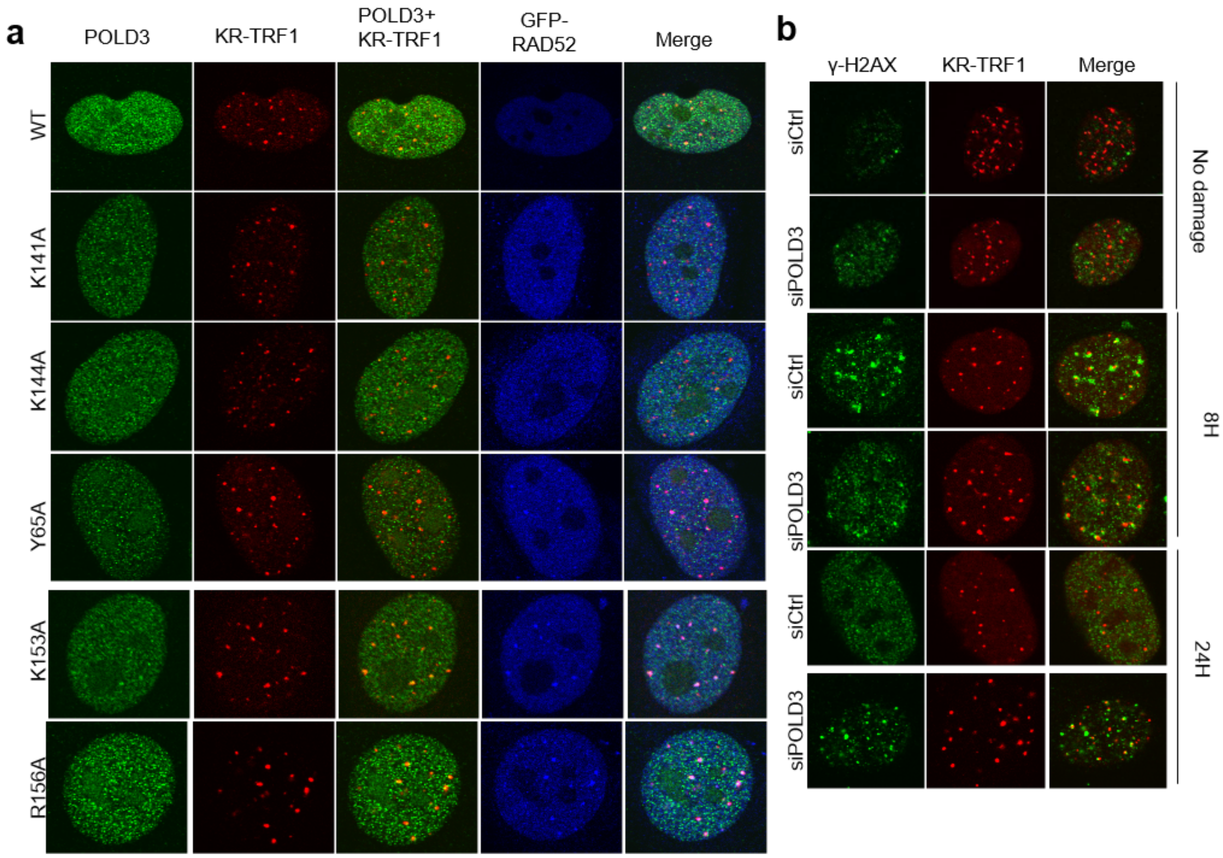

Supplementary Figure 7. RAD52 is required for the loading of POLD3 to damaged telomeres. **(a)** The recruitment of POLD3 to KR-TRF1 in U2OS RAD52 KO cells after transient expressing RAD52 mutants. **(b)** The clearance of γ-H2AX after POLD3 knockdown by siRNA

Supplementary Table 1:

Sequence-Based Primer	
AD-R464A-Rev	CCTTAACGCCTGCTTATAATAATCTTCATC
AD-R464A-For	AAGCAGGCGTTAAGGAGATGGAATAAACTG
AD-RR466, 467AA-Rev	ATTCCAAGCGGCTAACCGCTGCTTATAATAATC
AD-RR466, 467AA-For	CGGTTAGCCGCTTGGAATAAACTGAGACTGCAG
AD-3RA-Rev	ATTCCAAGCGGCTAACGCCTGCTTATAATAATCTTCATC
AD-K470A-For	TGGAATGCGCTGAGACTGCAGGACAAAGAG
AD-R472A-Rev	CTGCAGAGCCAGTTTATTCCATCTCCTTAAC
AD-R472A-For	AAACTGGCTCTGCAGGACAAAGAGAAACGTC
RAD52 K141A-For	TTGGAGGCCGCAAGGAAGGAGGCGGTGACAGACGGGC
RAD52 K141A-Rev	CGCCTCCTTCCTTGCGGCCTCCAAAGATAAAGCCTTG
RAD52 K144A-For	TTGGAGAAGGCAAGGGCCGAGGCGGTGACAGACGGGC
RAD52 K144A-Rev	CGCCTCGGCCCTTGCCTTCTCCAAAGATAAAGCCTTG
RAD52 Y65A-For	TAAGTAGCGCCATGGCTGGCGGAGGCCAGA
RAD52 Y65A- Rev	GCCAGCCATGGCGCTACTTATGTATTCTGGGC
RAD52 K152A-For	ACGGGCTGGCGCGAGCCCTCAGGAGTTTT
RAD52 K152A-Rev	AGGGCTCGCGCCAGCCCGTCTGTCACC
RAD52 K153A-For	GGCTGAAGGCAGCCCTCAGGAGTTTTGGGAA
RAD52 K153A-Rev	CTGAGGGCTGCCTTCAGCCCGTCTGTCACC
RAD52 K156A-For	CGAGCCCTCGCGAGTTTTGGGAATGCACTTGG
RAD52 K156A-Rev	CCAAAACTCGCGAGGGCTCGCTTCAGCCCG

Supplementary Table 2:

Sequence-Based Reagents	Source
TERRA FISH probe: (TAACCC)7-Alexa488-30	Integrated DNA Technologies
LNA gapmer Scr: 5’-CACGTCTATACACCAC-3’	Exiqon
LNA gapmer TERRA: 5’-TAACCCTAACCCTAAC-3’	Exiqon
TERRA probe for Northern blot : 5’-TAACCCTAACCC TAACCCTAACCCTAACCC-3’	Integrated DNA Technologies
GAPDH probe for Northern blot : 5’-GTAGACCCACGACA TACTCAGCACCGGCCTCACCCCATT-3’	Integrated DNA Technologies
Oligo 1：5’-ATCATCACCATAACGTCGATGTATCAA CTTCGATTAGTCACACCAATTAA-3’	Integrated DNA Technologies
Oligo 2: 5’-UUAAUUGGUGUGACUAAUCGAAGUUGAUA CAUCGACGUUAUGGUGAUGAU-3’	Integrated DNA Technologies
